# Supplementary material for: Synthesis and Evaluation of Trypanocidal Activity of Chromane-Type Compounds and Acetophenones
Source: Molecules. 2021 Nov 23;26(23):7067. doi: 10.3390/molecules26237067 (PMC8658963; doi:10.3390/molecules26237067)
Supplement: Supplementary file 1 [file molecules-26-07067-s001.zip › molecules-1344261-supplementary.pdf]

Supplemental Material

Synthesis and Evaluation of Trypanocidal Activity of Chromane-type Compounds and Acetophenones

Luis A González[1], Sara Robledo[2], Yulieth Upegui[2], Gustavo Escobar[1], Wiston Quiñones[1]*

1. Grupo de Química Orgánica de Productos Naturales, QOPN, Instituto de Química, Facultad de Ciencias Exactas y Naturales, Universidad de Antioquia, Calle 70 No. 52–21, Medellín A. A 1226, Colombia.

2. PECET-Facultad de Medicina, Universidad de Antioquia, Calle 70 # 52-21, Medellín Colombia

*Correspondence: Wiston Quiñones, Grupo de Química Orgánica de Productos Naturales, QOPN, Instituto de Química, Facultad de Ciencias Exactas y Naturales, Universidad de Antioquia, Calle 70 No. 52–21, Medellín A. A 1226, Colombia

Email: wiston.quinones@udea.edu.co

1-(2,4-Dihydroxyphenyl)ethan-1-one (**5**)

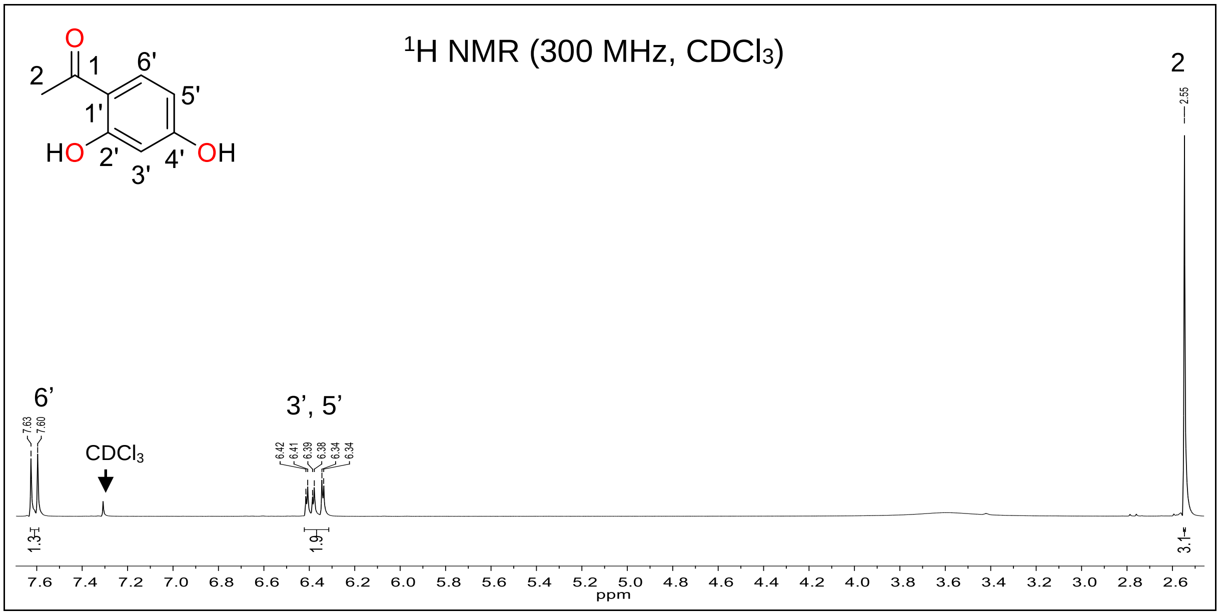

^{1}H NMR (**5**)

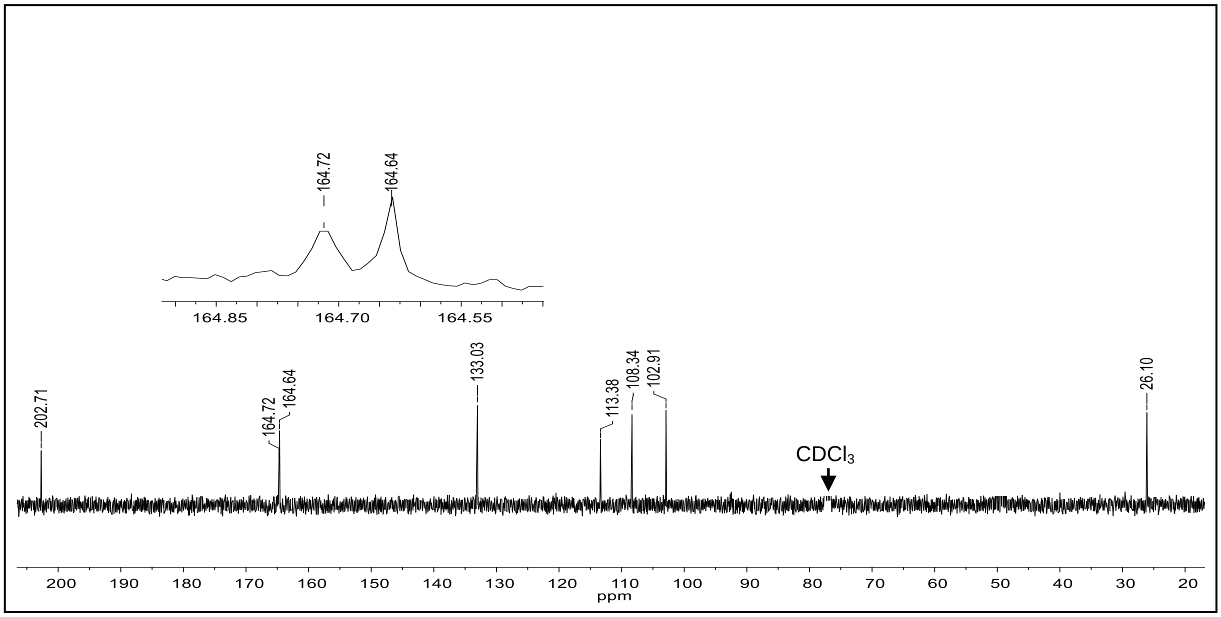

^{13}C NMR (**5**)

1-(2,5-Dihydroxyphenyl)ethan-1-one (**6**)

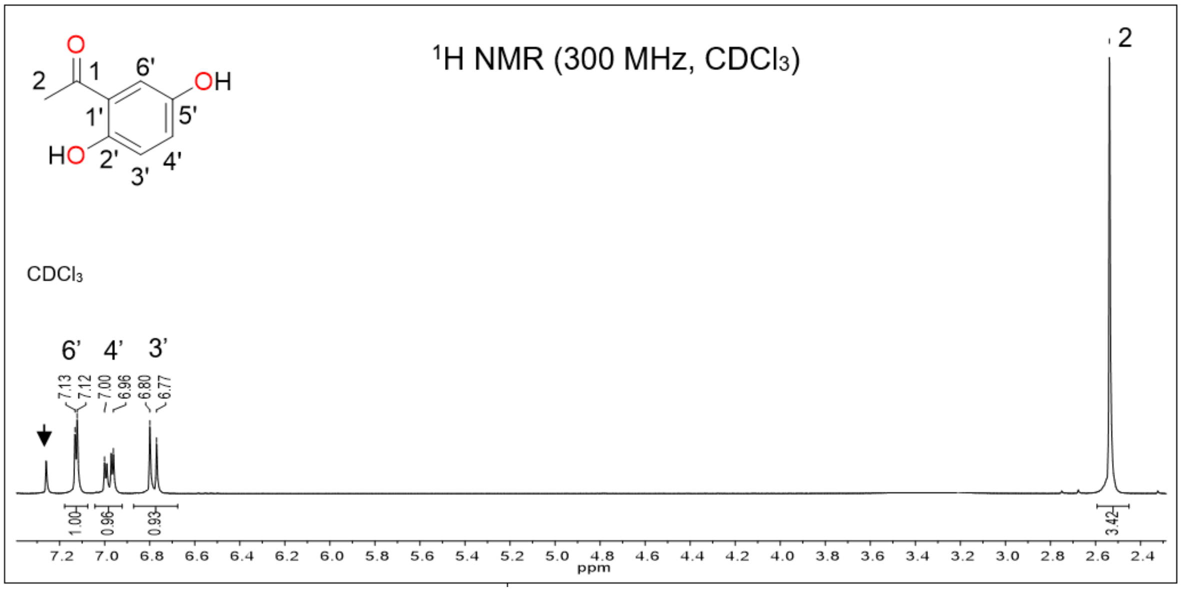

^{1}H NMR (**6**)

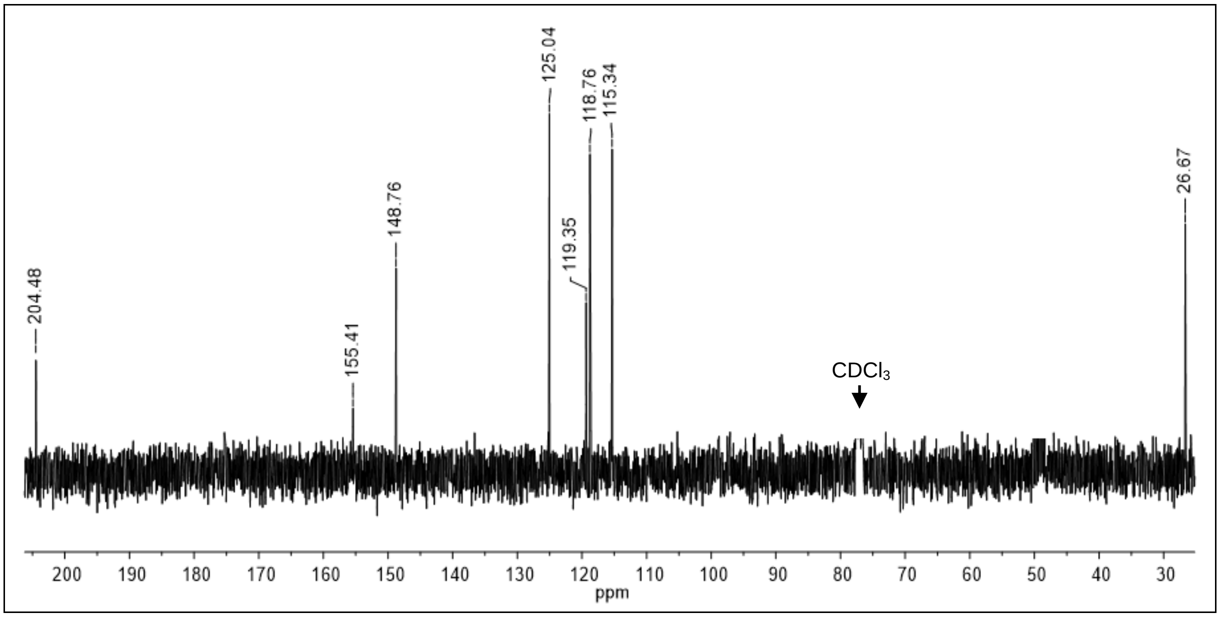

^{13}C NMR (**6**)

1-(2,6-Dihydroxyphenyl)ethan-1-one (**7**)

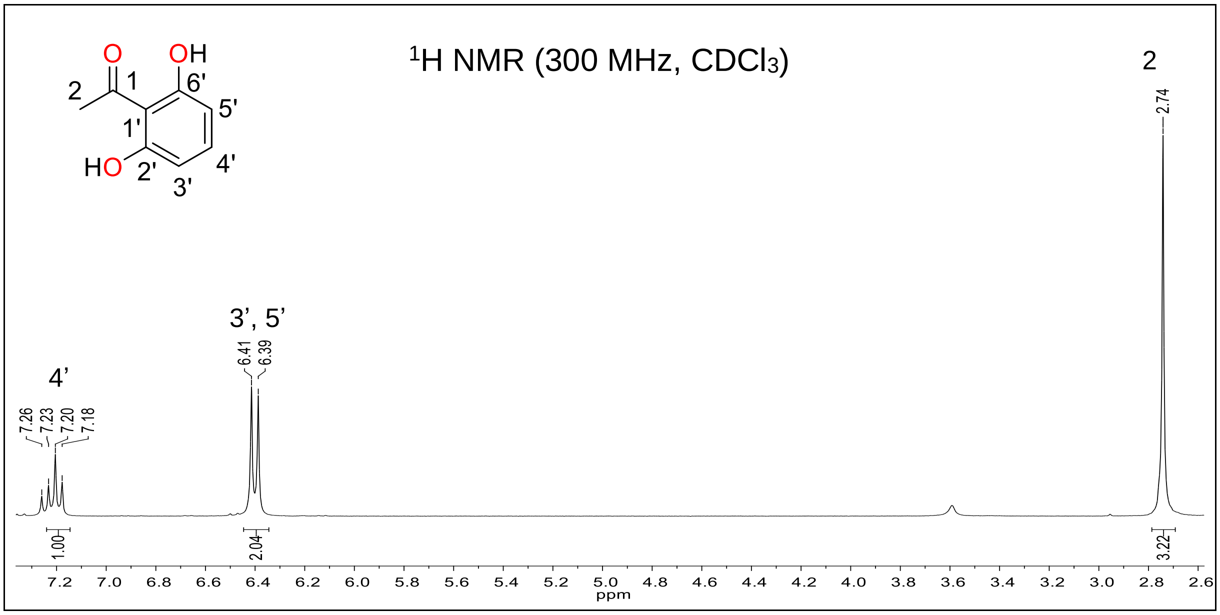

^{1}H NMR (**7**)

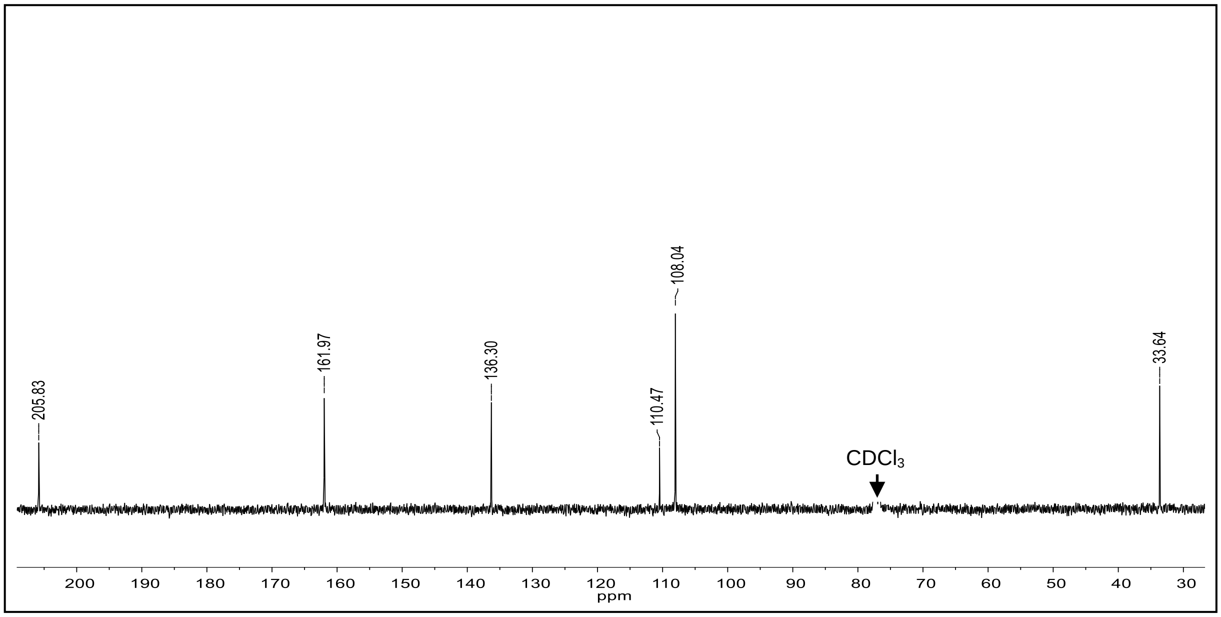

^{13}C NMR (**7**)

1-(2,3,4-Trihydroxyphenyl)ethan-1-one (**8**)

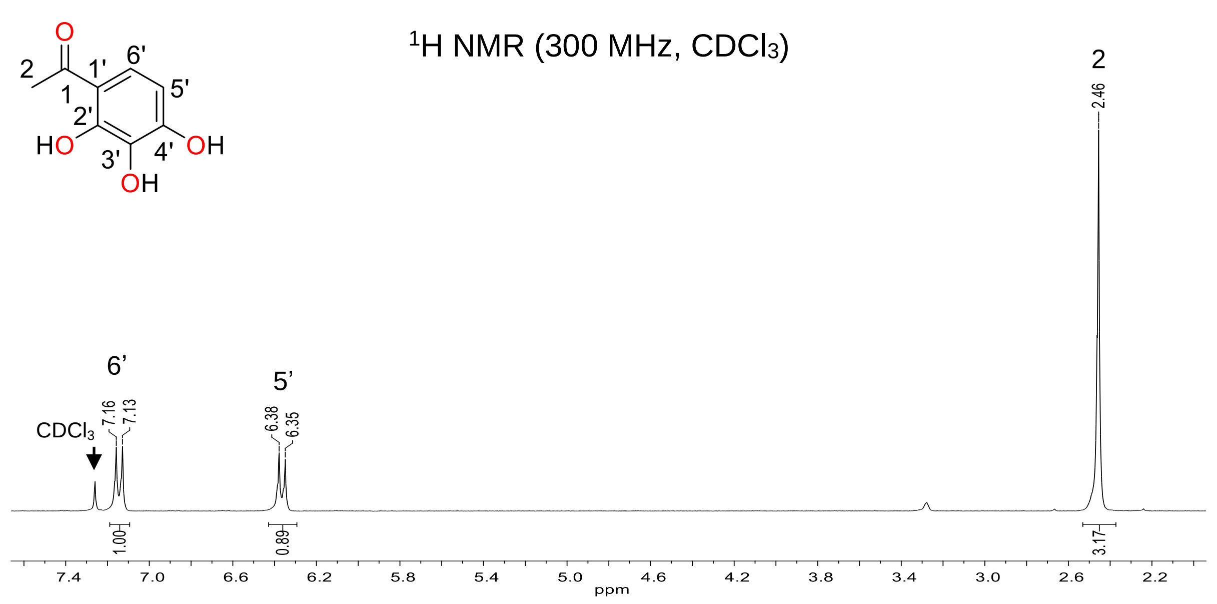

^{1}H NMR (**8**)

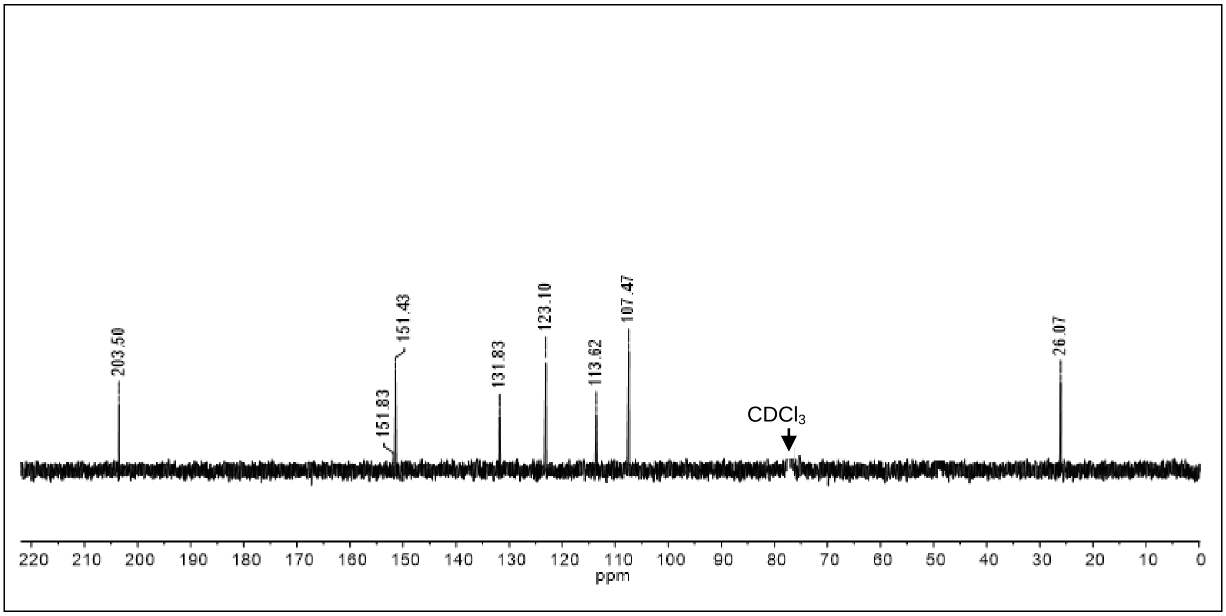

^{13}C NMR (**8**)

1-(2,4,6-Trihydroxyphenyl)ethan-1-one (**9**)

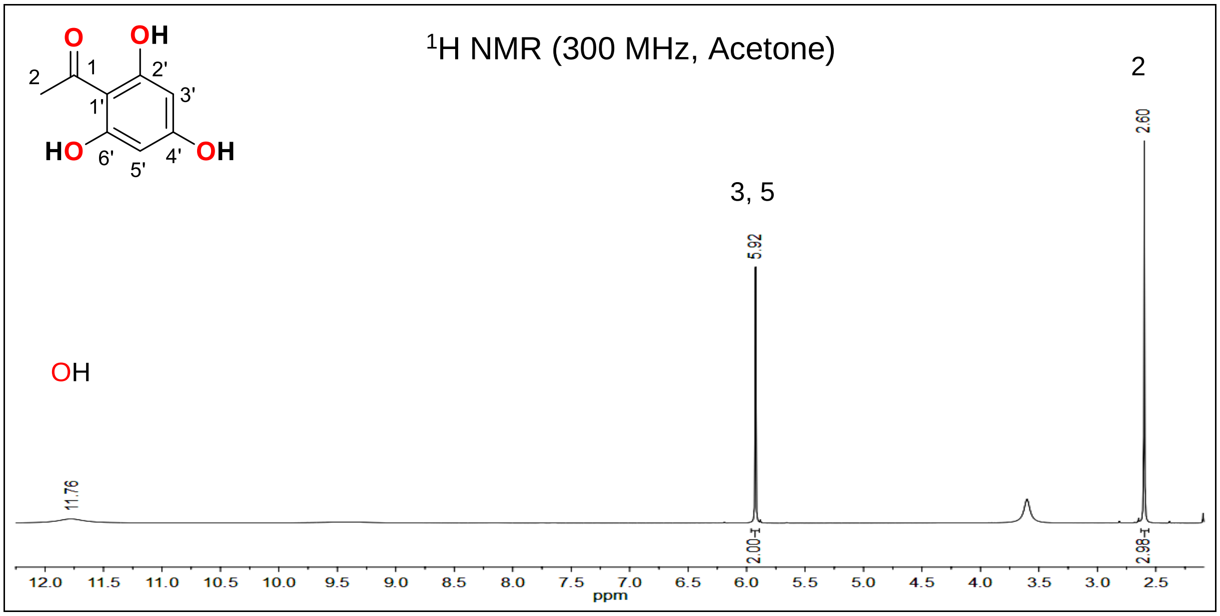

^{1}H NMR (**9**)

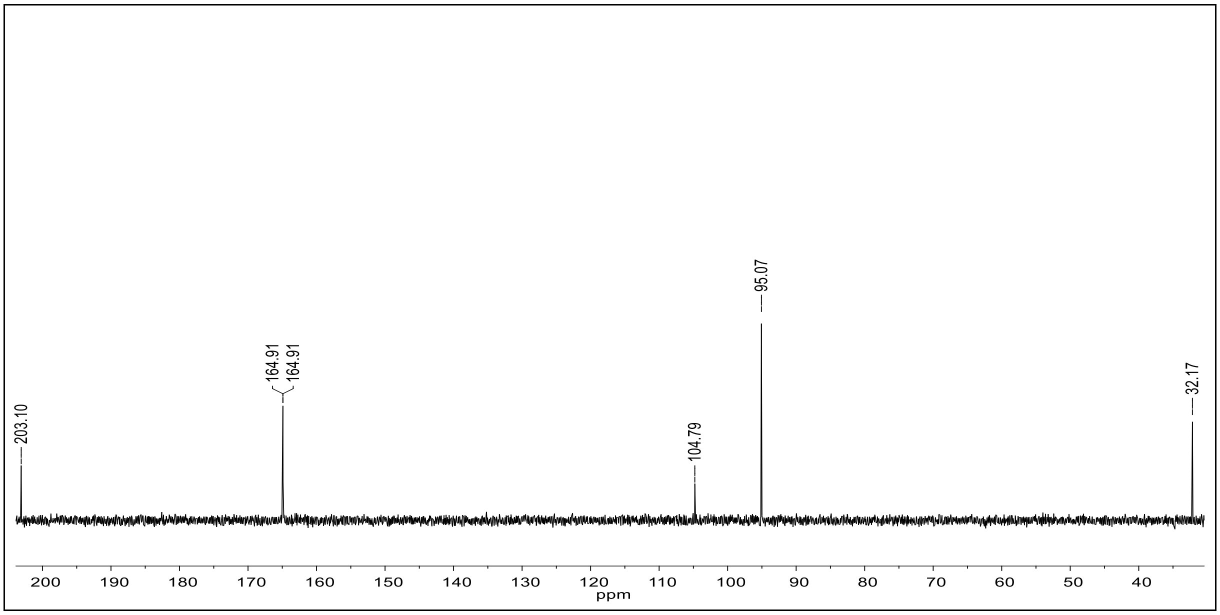

^{13}C NMR (**9**)

7-Hydroxy-4H-chromen-4-one (**10**)

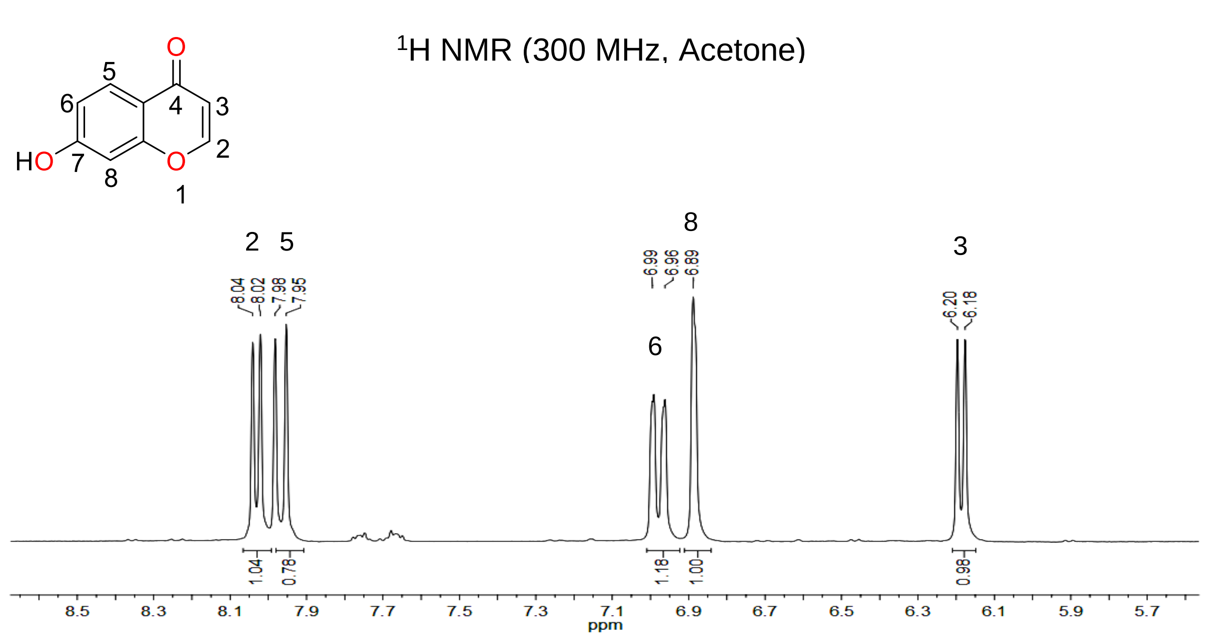

^{1}H NMR (**10**)

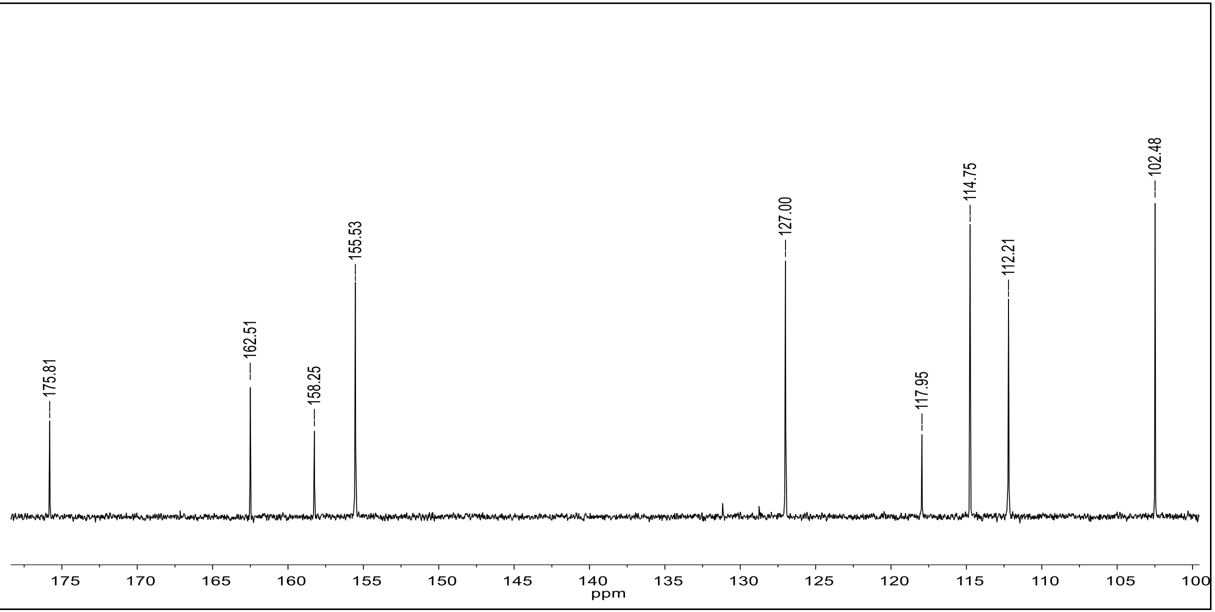

^{13}C NMR (**10**)

6-Hydroxy-4H-chromen-4-one (**11**)

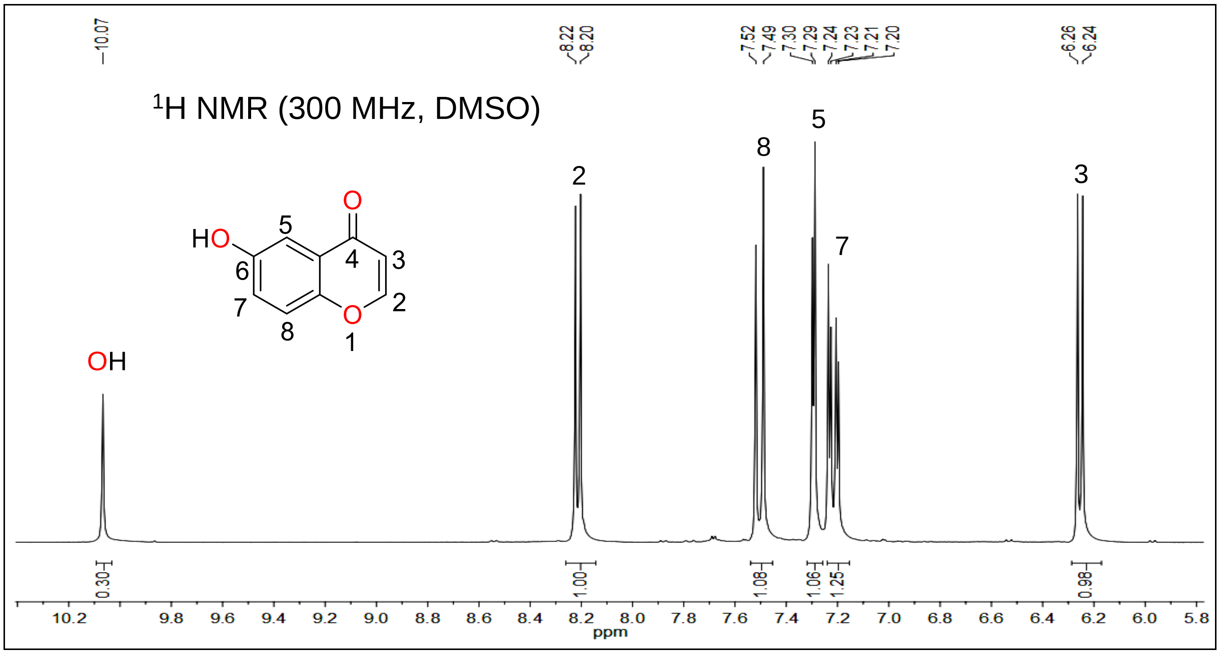

^{1}H NMR (**11**)

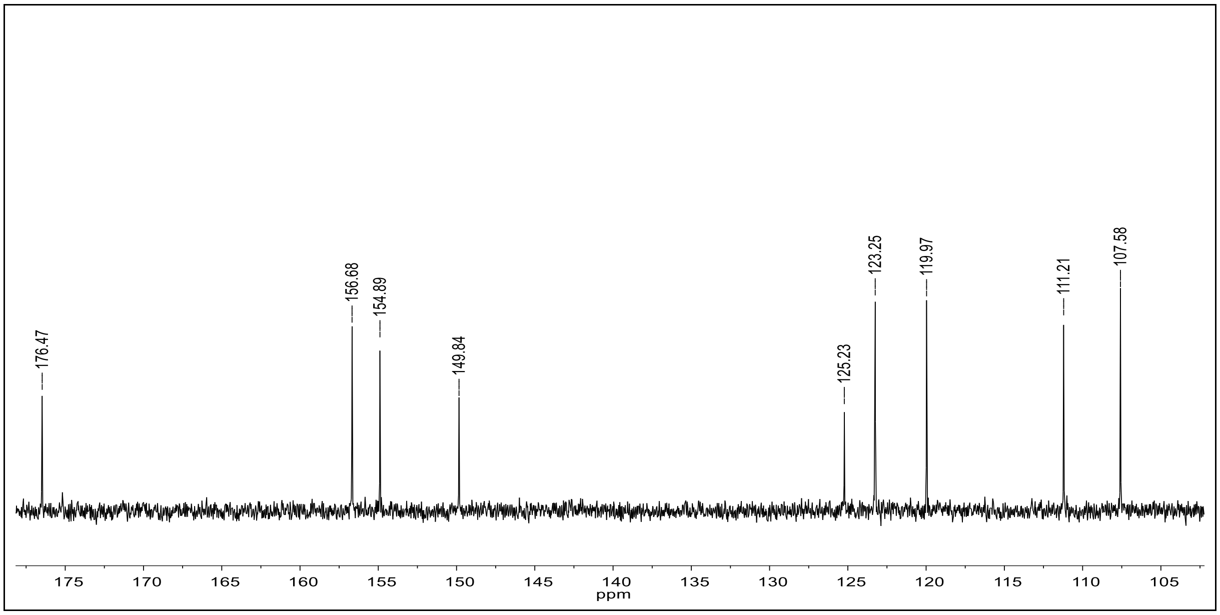

^{13}C NMR (**11**)

5-Hydroxy-4H-chromen-4-one (**12**)

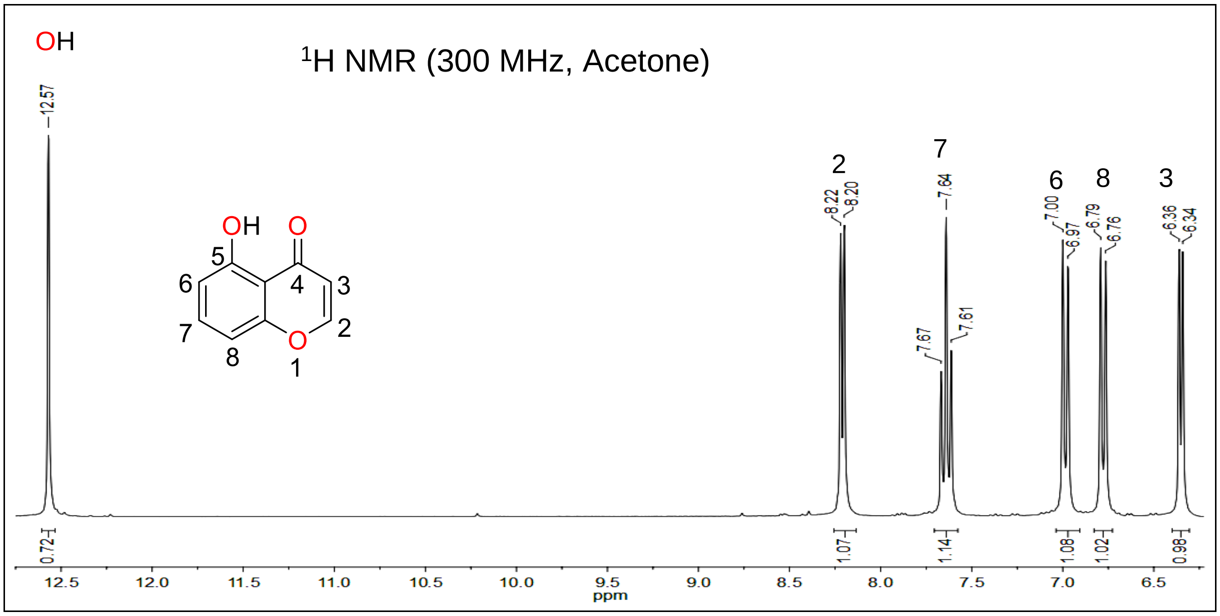

^{1}H NMR (**12**)

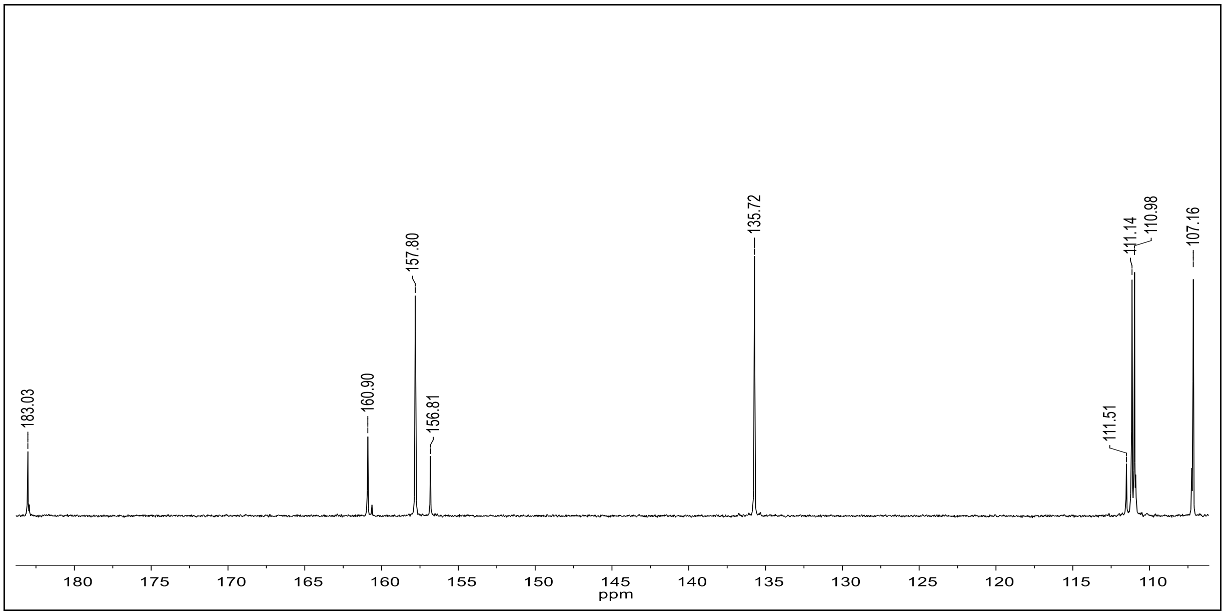

^{13}C NMR (**12**)

7,8-Dihydroxy-4H-chromen-4-one (**13**)

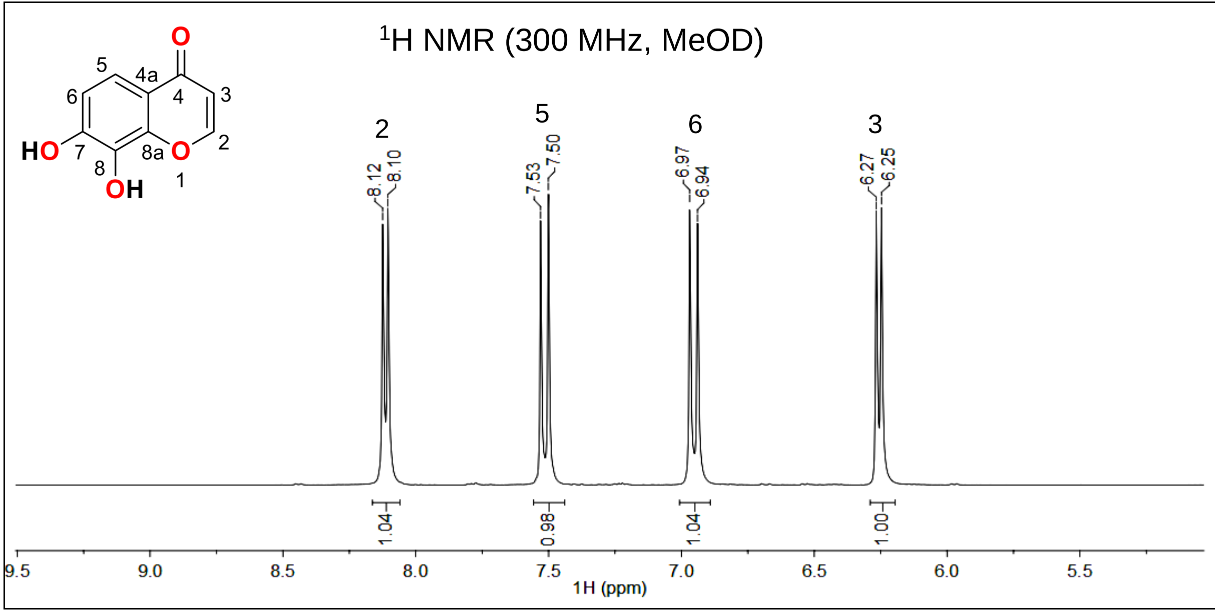

^{1}H NMR (**13**)

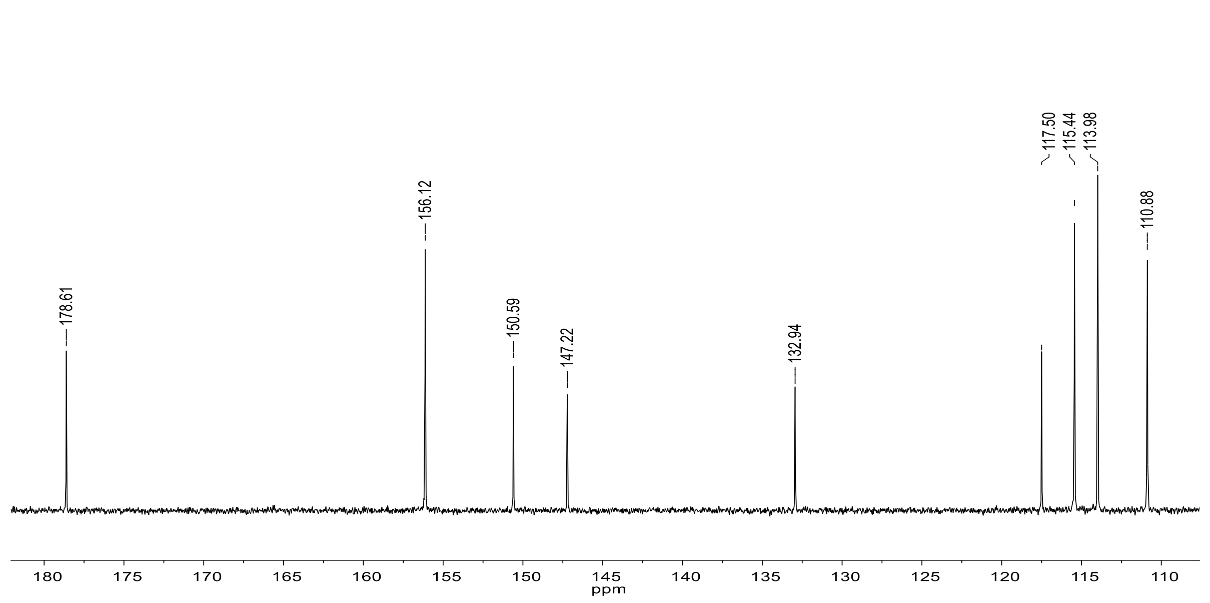

^{13}C NMR (**13**)

1-(2,4-Dihydroxy-5-(3-methylbut-2-en-1-yl)phenyl)ethan-1-one (**14**)

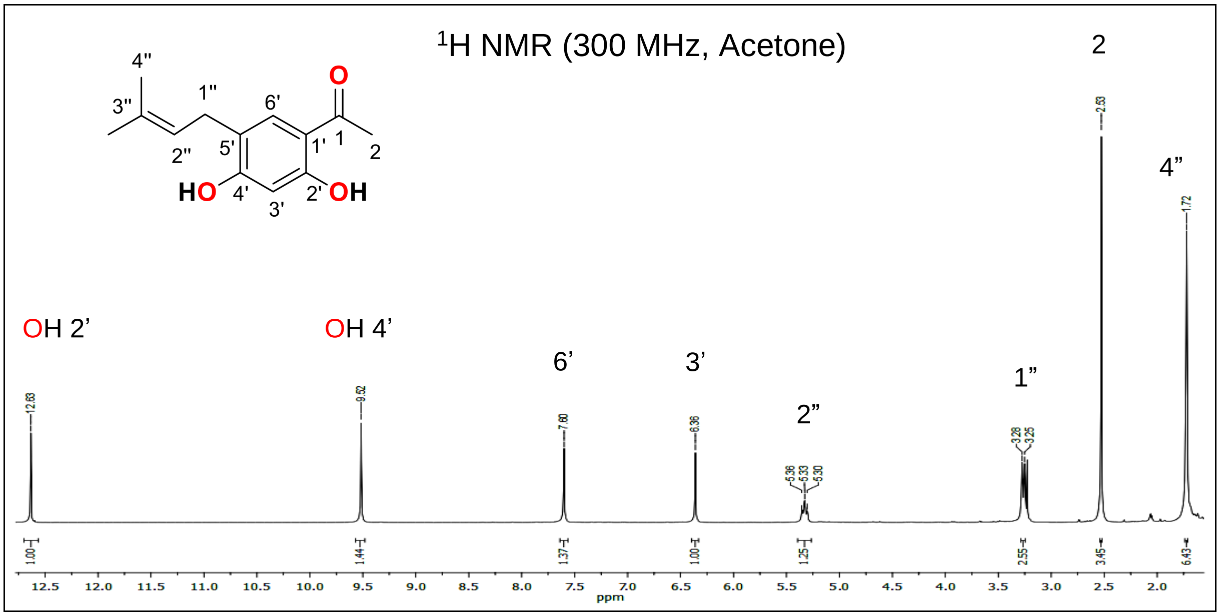

¹H NMR (**14**)

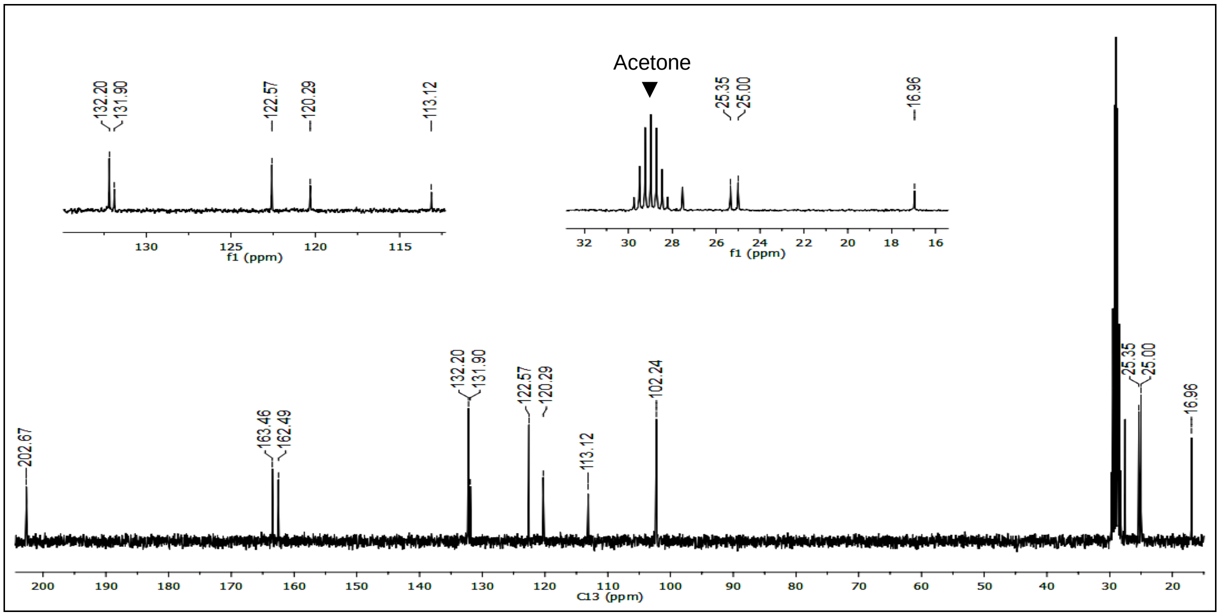

¹³C NMR (**14**)

1-(2,4-Dihydroxy-3-(3-methylbut-2-en-1-yl)phenyl)ethan-1-one (**15**)

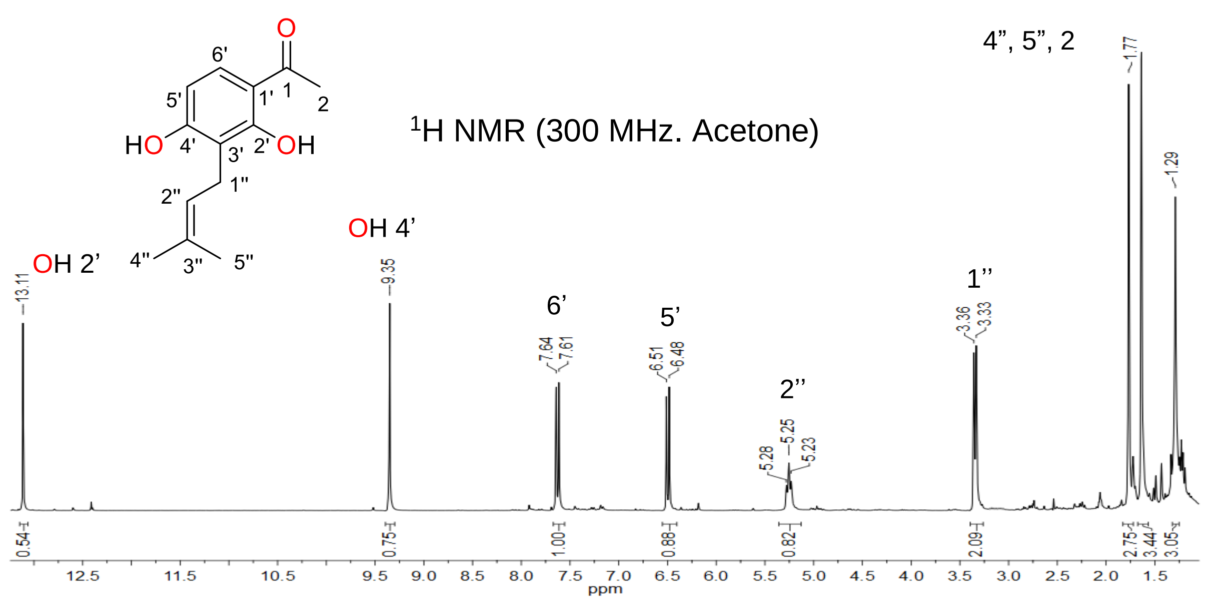

^{1}H NMR (**15**)

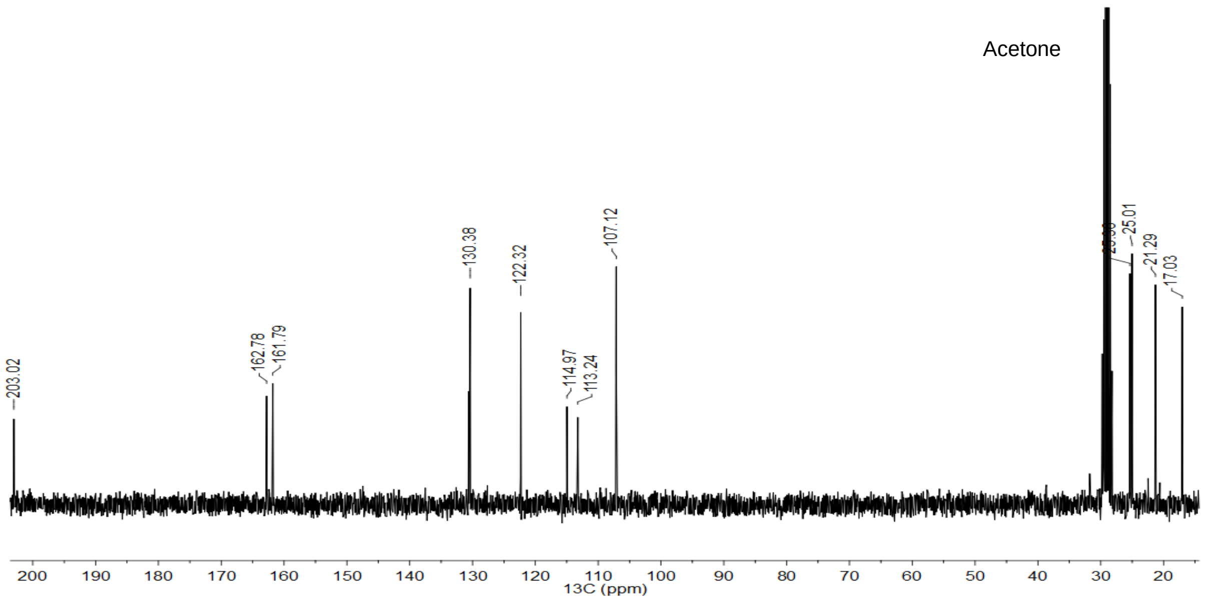

^{13}C NMR (**15**)

1-(2,6-Dihydroxy-3-(3-methylbut-2-en-1-yl)phenyl)ethan-1-one (**16**)

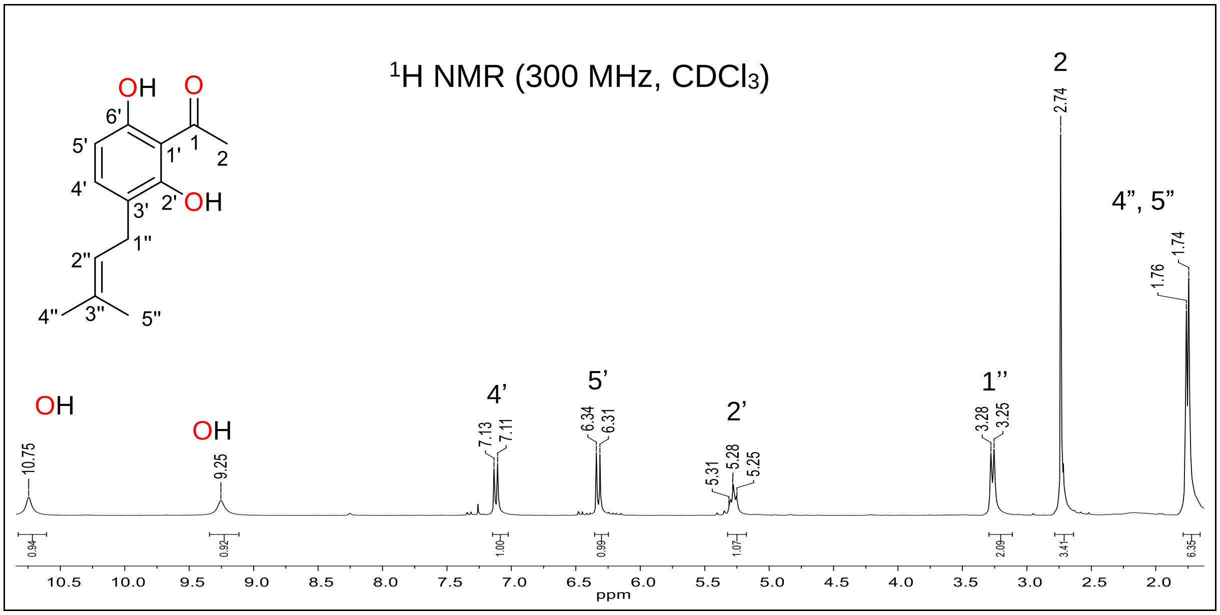

^{1}H NMR (**16**)

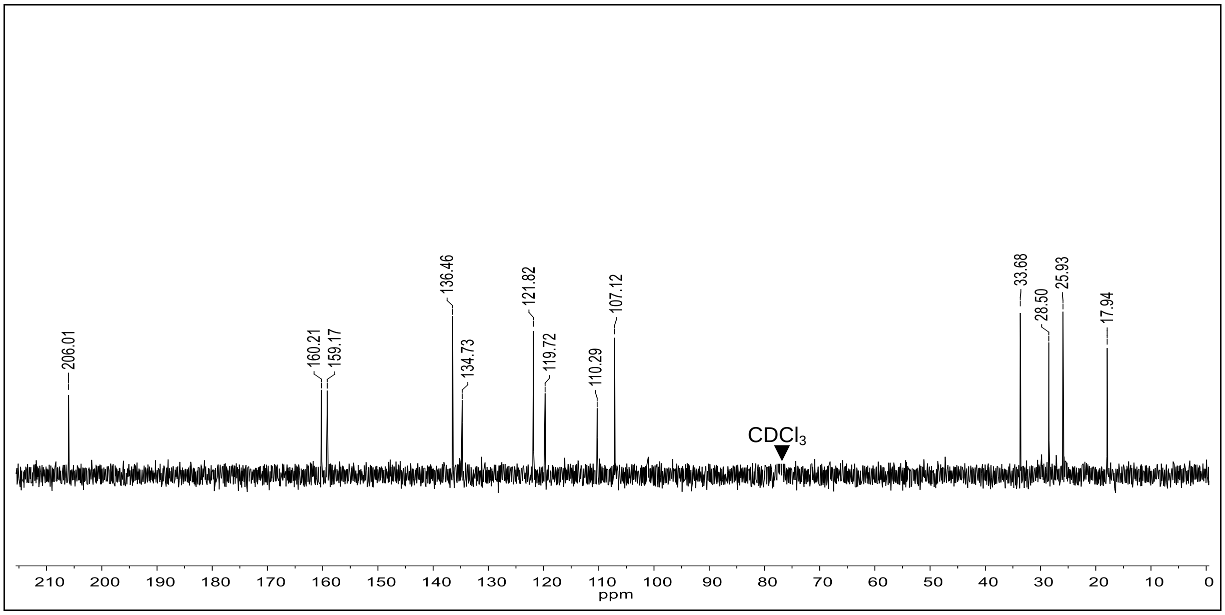

^{13}C NMR (**16**)

1-(2,4,6-Trihydroxy-3-(3-methylbut-2-en-1-yl)phenyl)ethan-1-one (**17**)

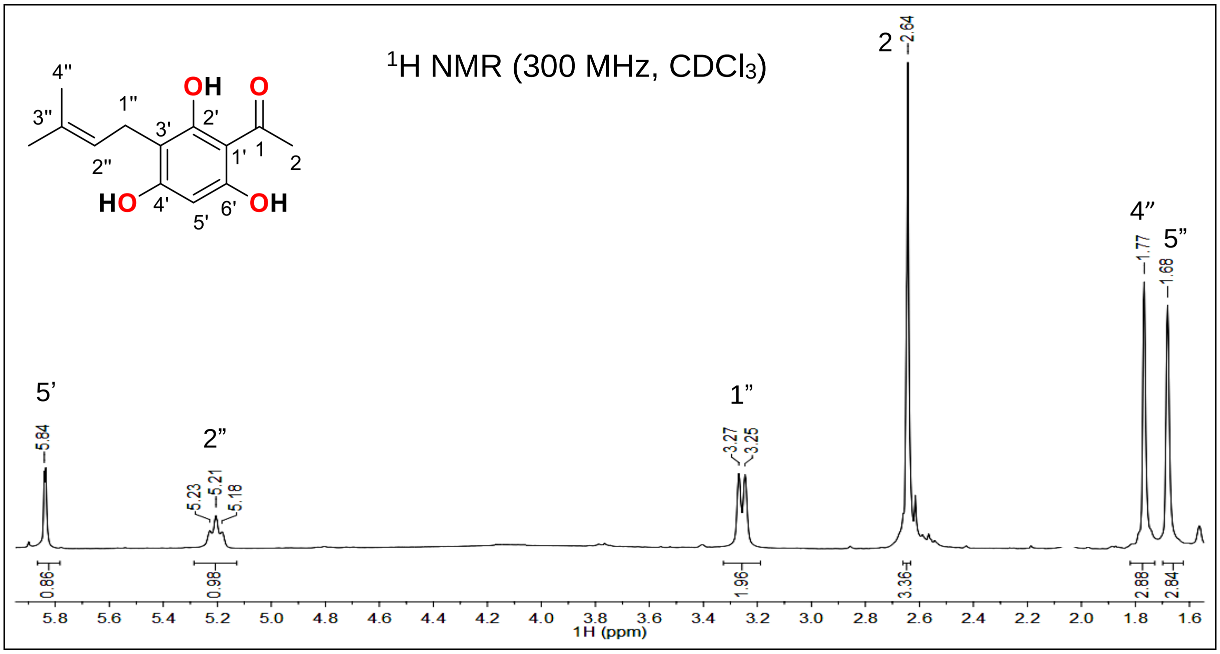

^{1}H NMR (**17**)

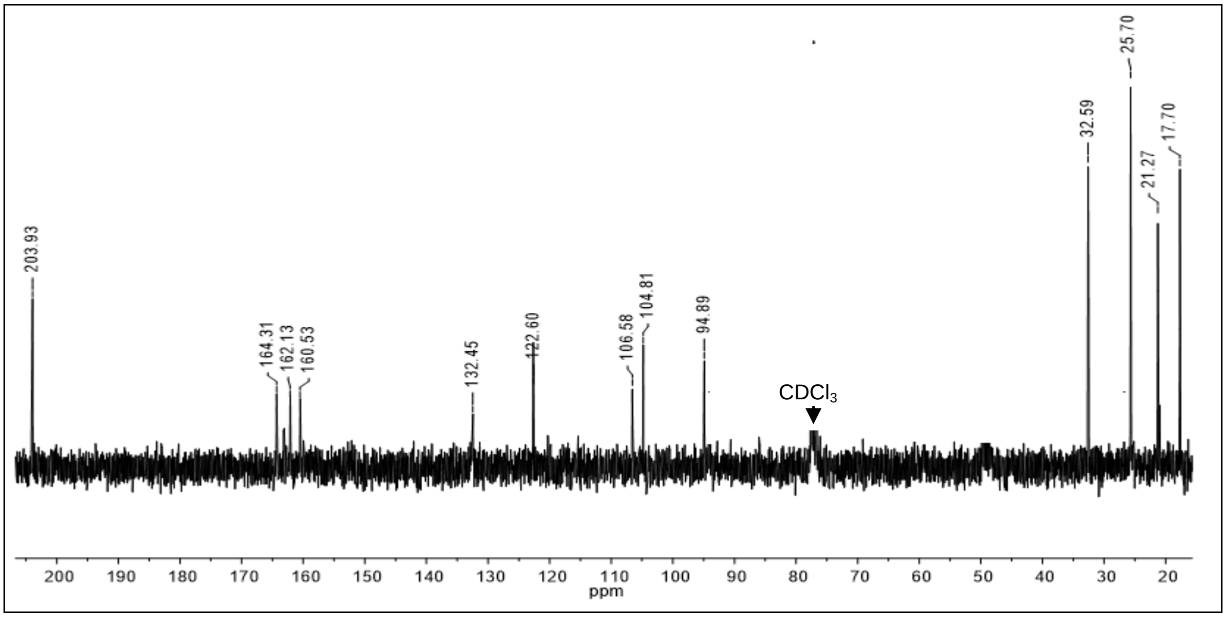

^{13}C NMR (**17**)

1-(2,3,4-Trihydroxy-5-(3-methylbut-2-en-1-yl)phenyl)ethan-1-one (**18**)

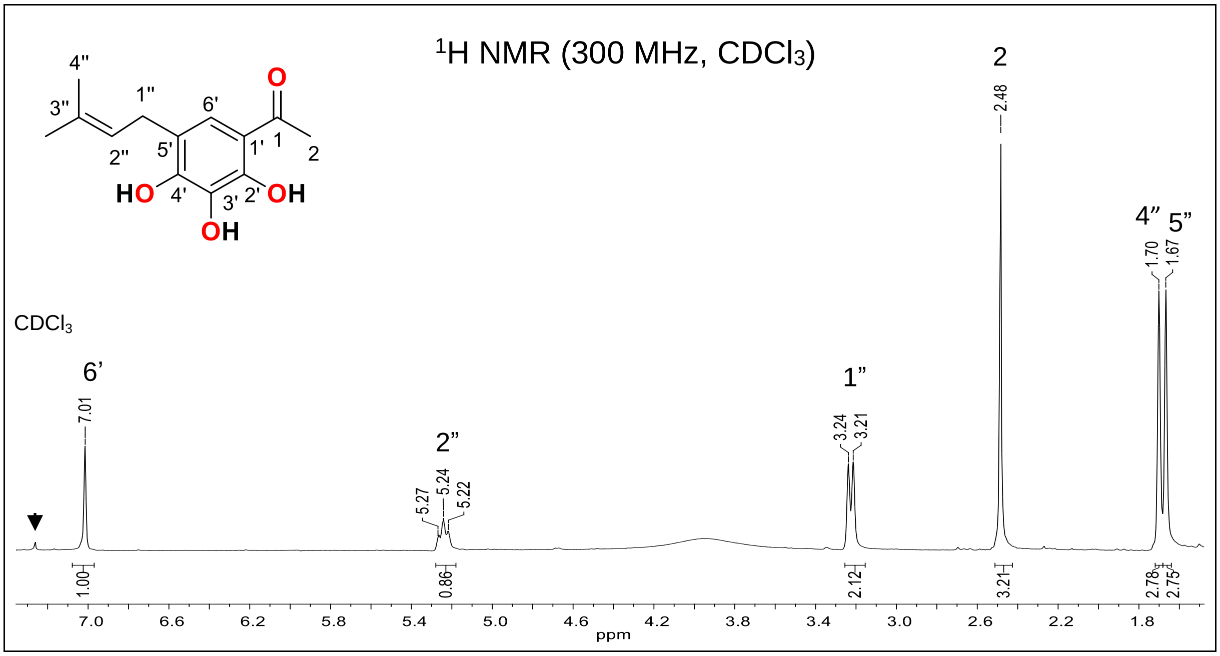

^{1}H NMR (**18**)

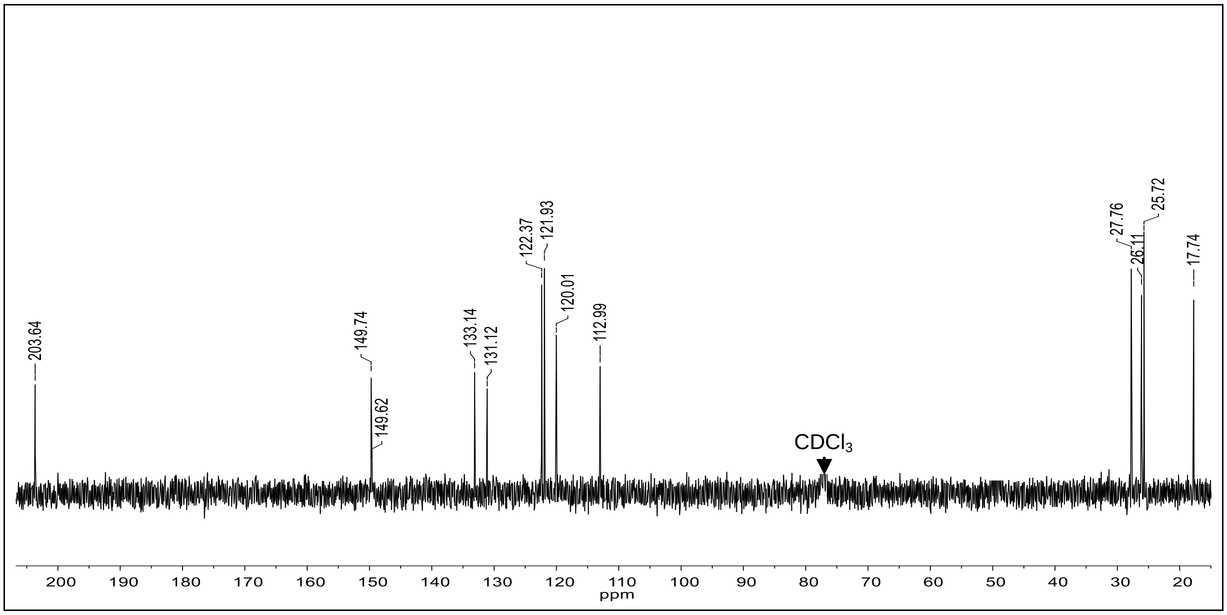

^{13}C NMR (**18**)

7-Hydroxy-6-(3-methylbut-2-en-1-yl)-4H-chromen-4-one (**23**)

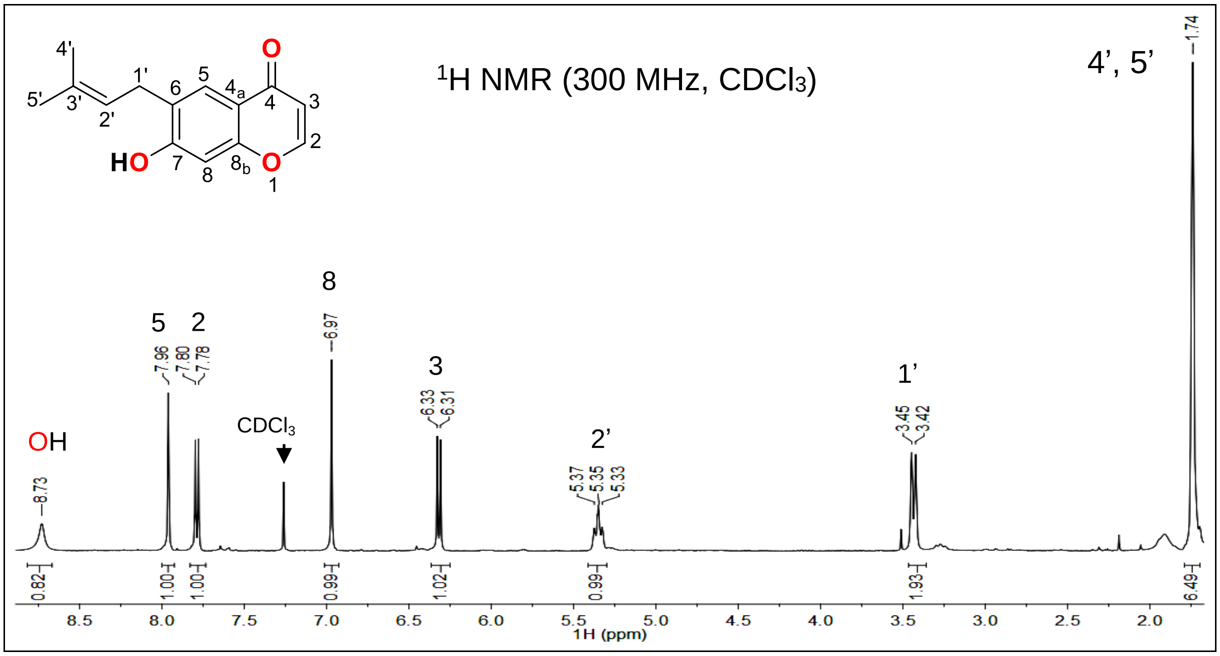

^{1}H NMR (**23**)

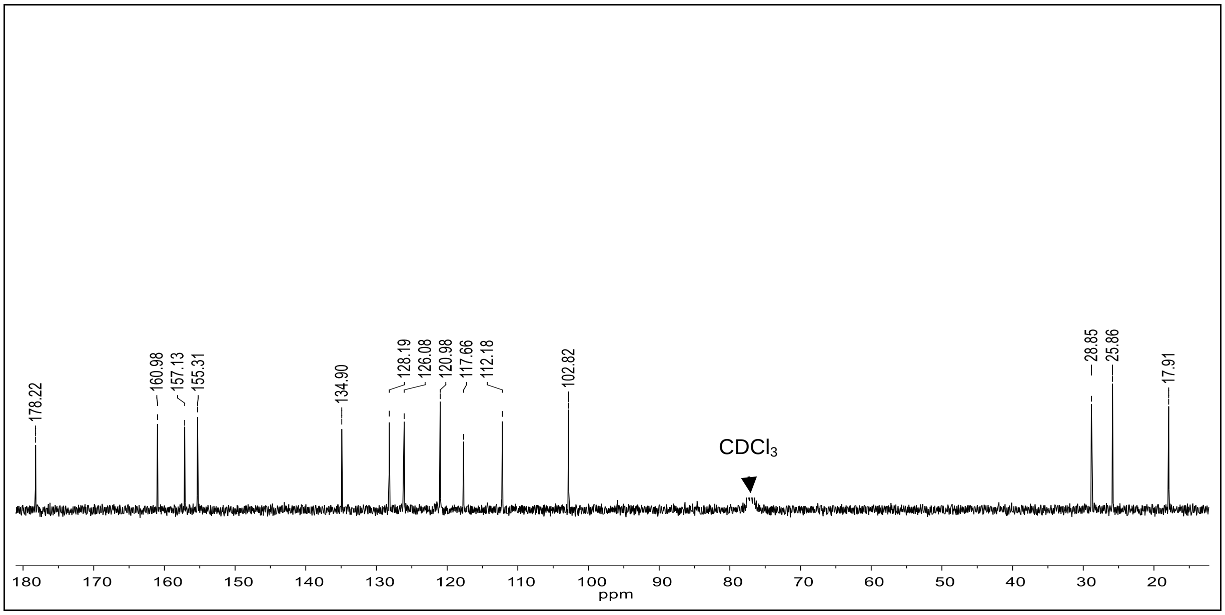

^{13}C NMR (**23**)

7-Hydroxy-8-(3-methylbut-2-en-1-yl)-4H-chromen-4-one (**24**)

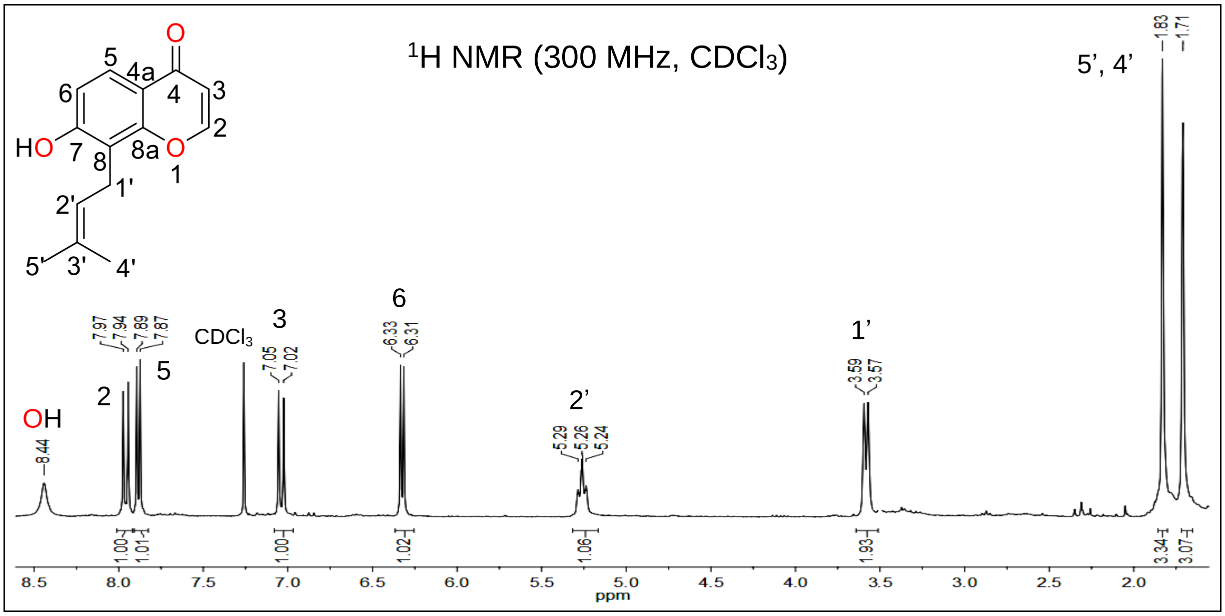

^{1}H NMR (**24**)

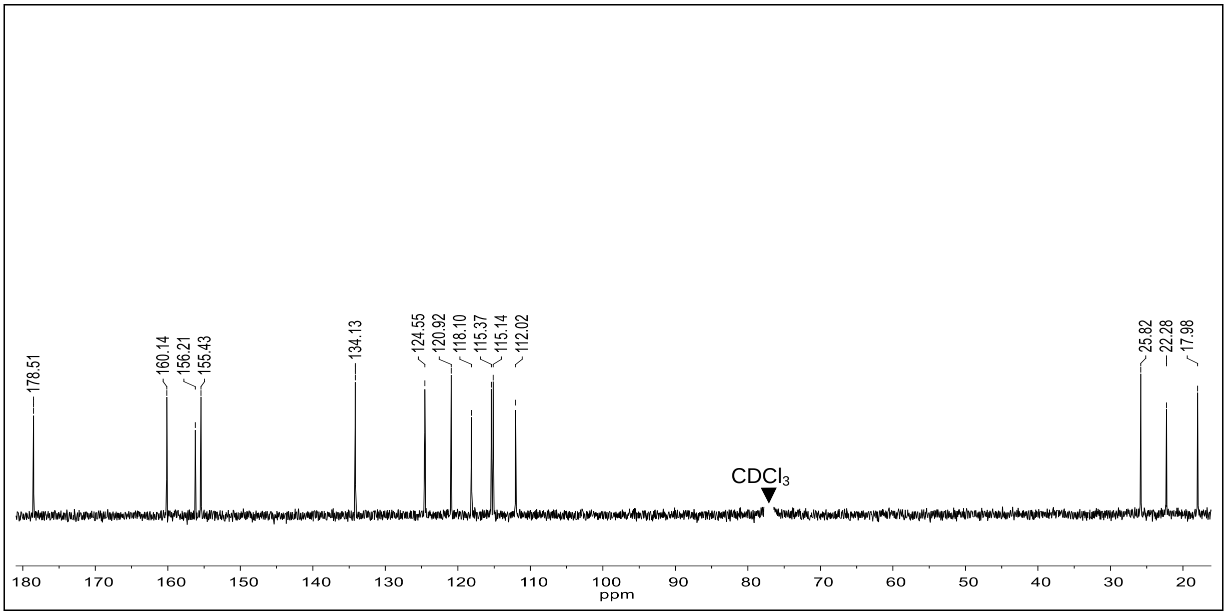

^{13}C NMR (**24**)

5-Hydroxy-6-(3-methylbut-2-en-1-yl)-4H-chromen-4-one (**25**)

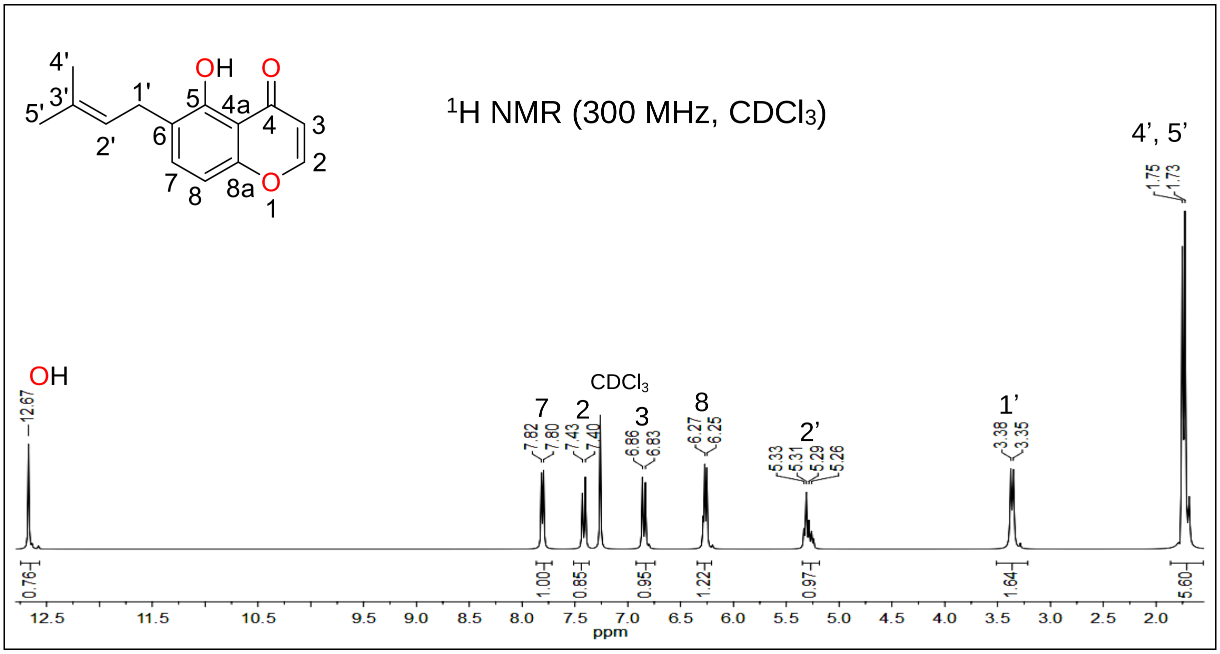

^{1}H NMR (**25**)

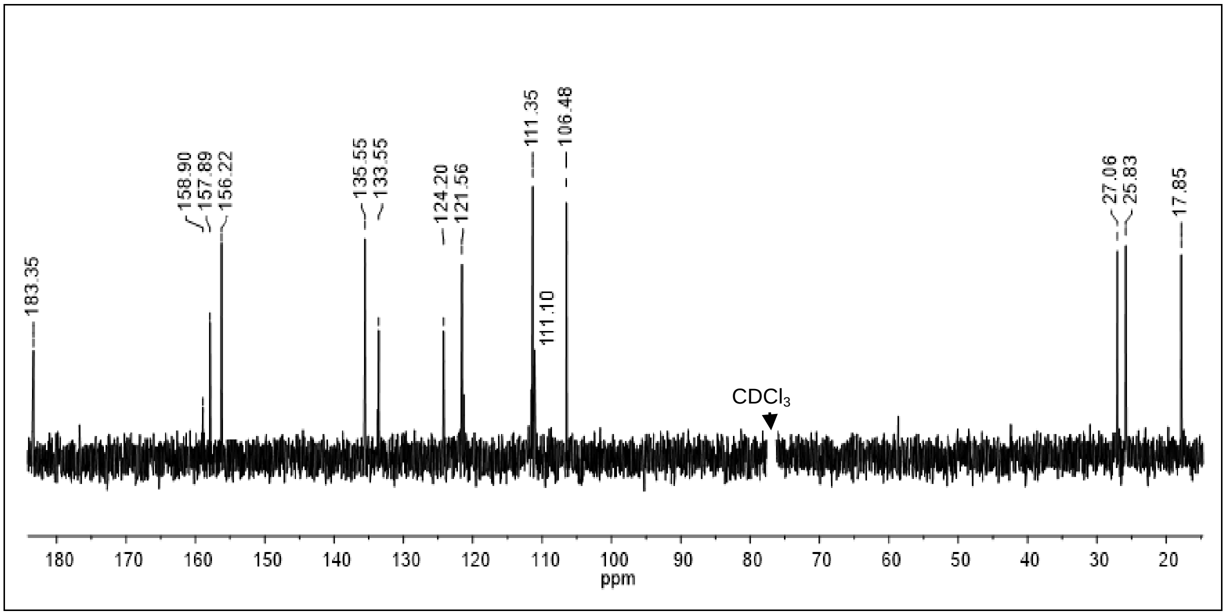

^{13}C NMR (**25**)

7,8-Dihydroxy-6-(3-methylbut-2-en-1-yl)-4H-chromen-4-one (**26**)

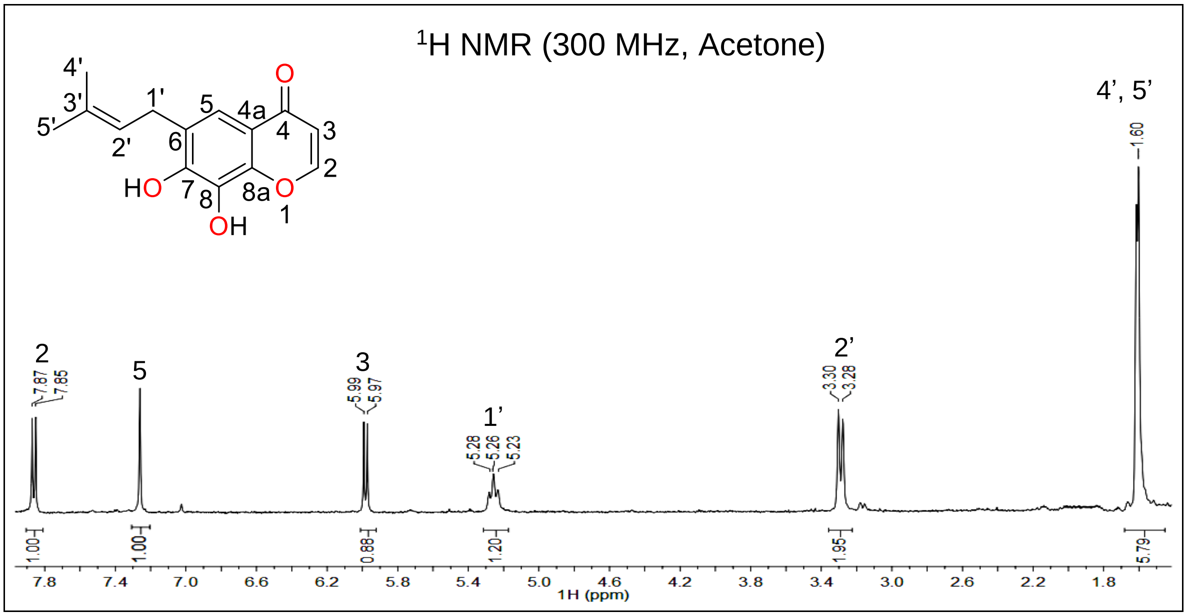

^{1}H NMR (**26**)

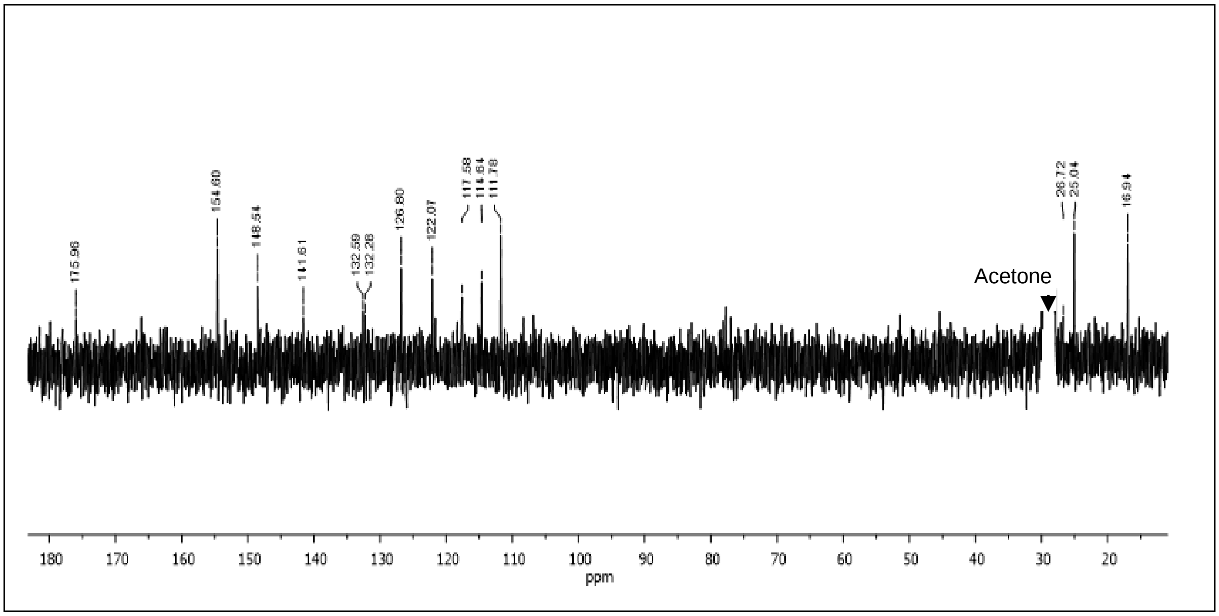

^{13}C NMR (**26**)

1-(2-Hydroxy-4-(methoxymethoxy)-5-(3-methylbut-2-en-1-yl)phenyl)-3-(4-methoxyphenyl)prop-2-en-1-one (**27**)

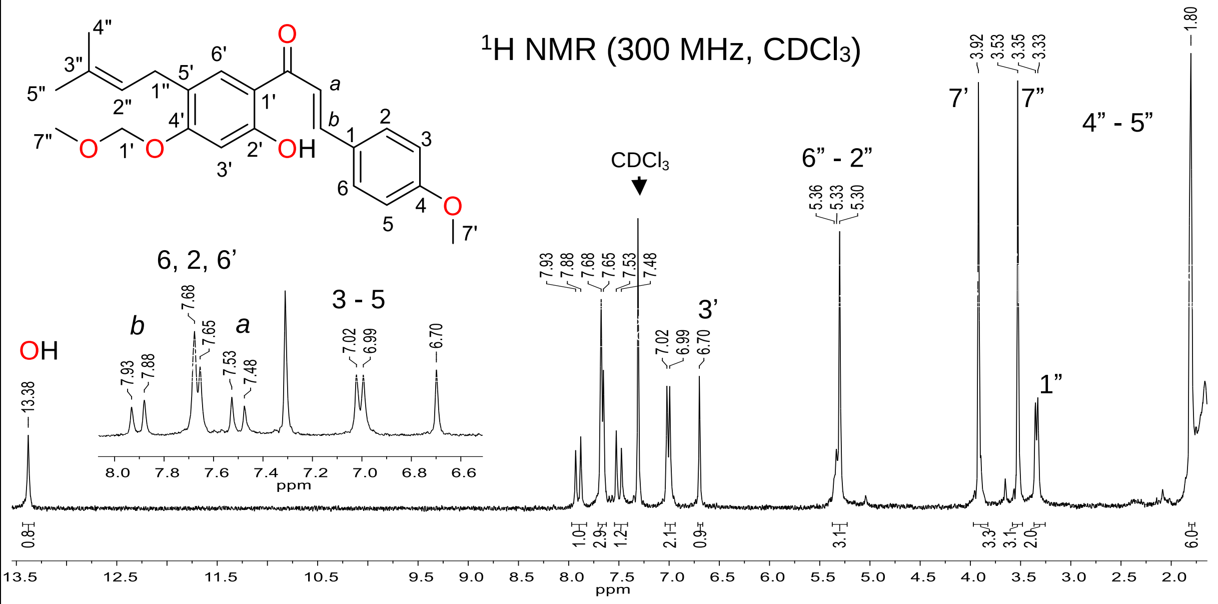

¹H NMR de (**27**)

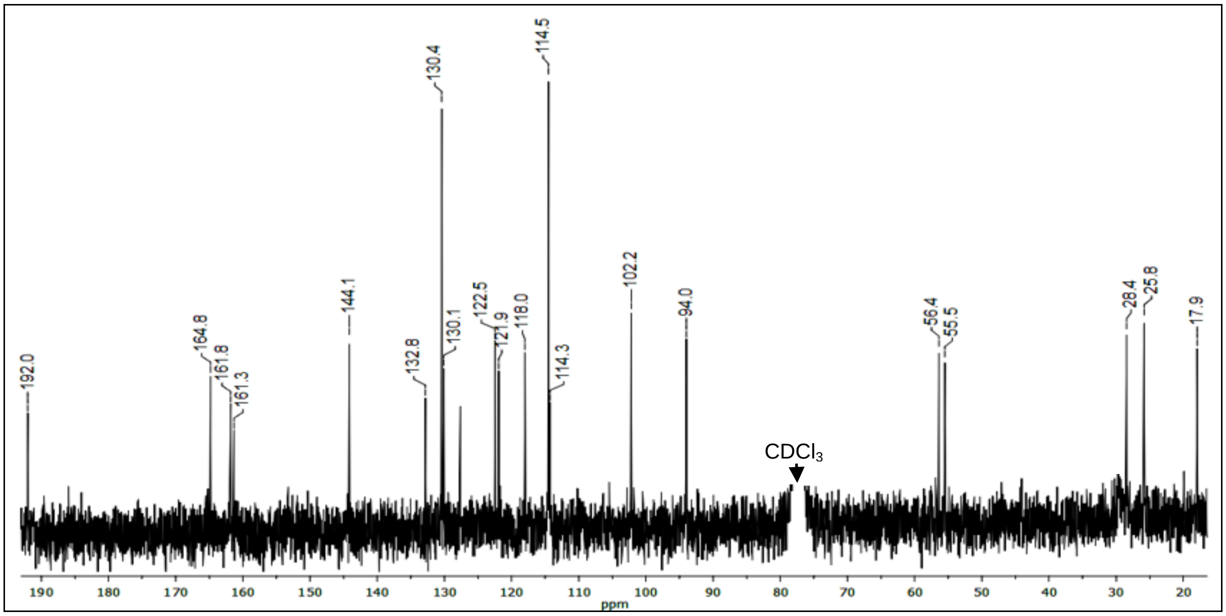

^{13}C NMR (**27**)

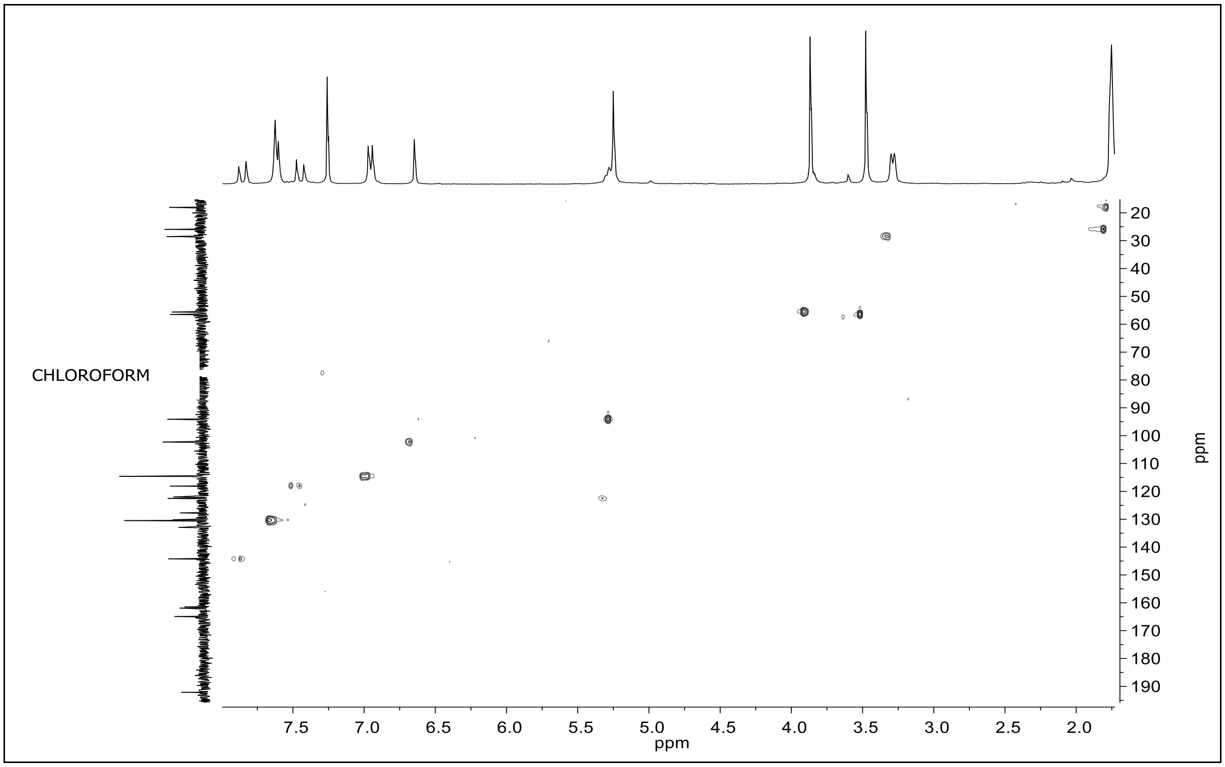

HMQC NMR (**27**)

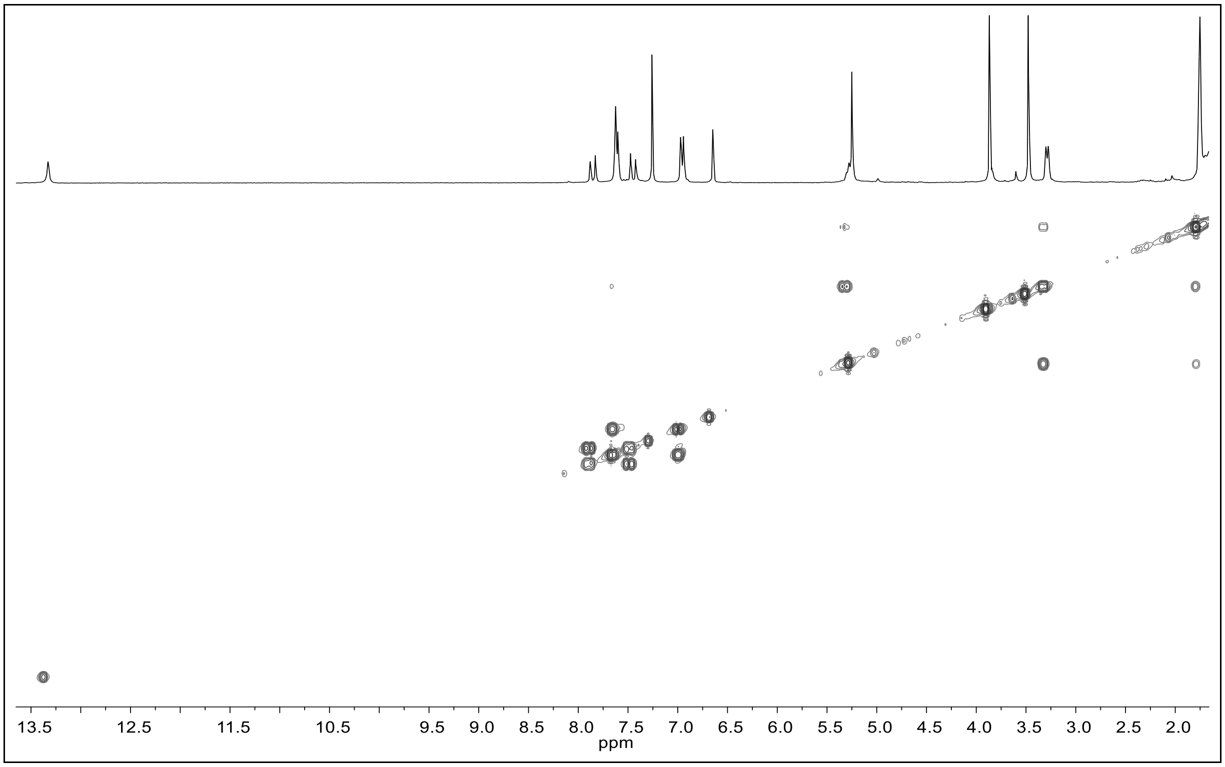

COSY NMR (**27**)

3-(4-Chlorophenyl)-1-(2-hydroxy-4-(methoxymethoxy)-5-(3-methylbut-2-en-1-yl)phenyl)prop-2-en-1-one (**28**)

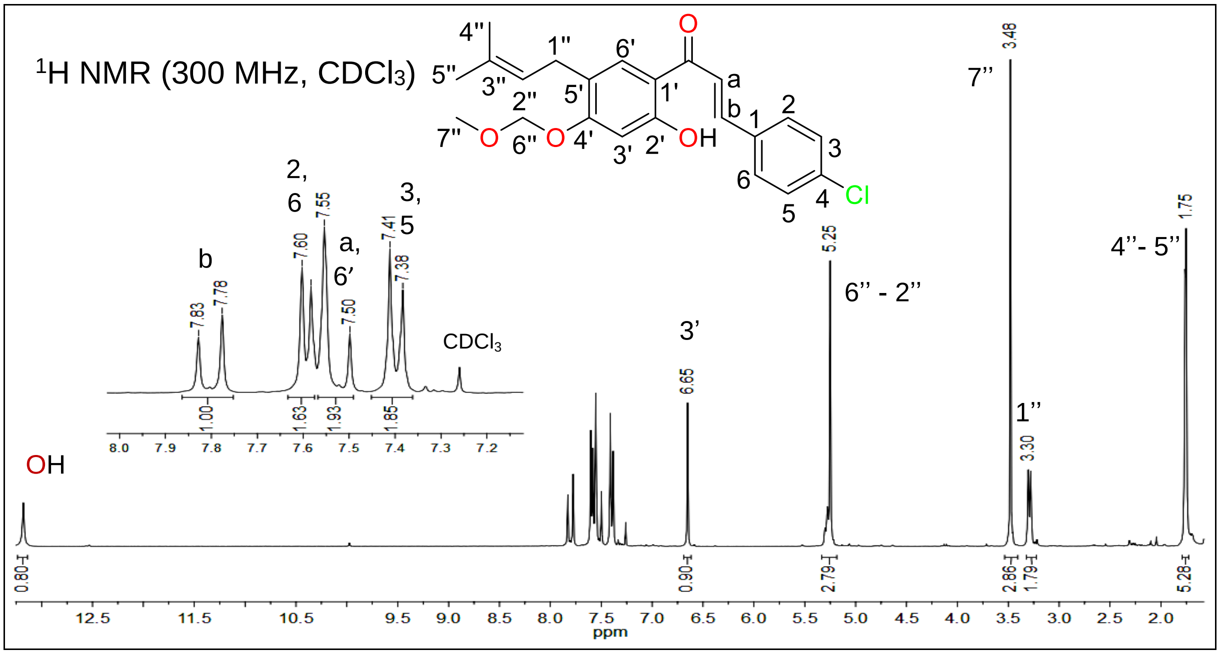

^{1}H NMR (**28**)

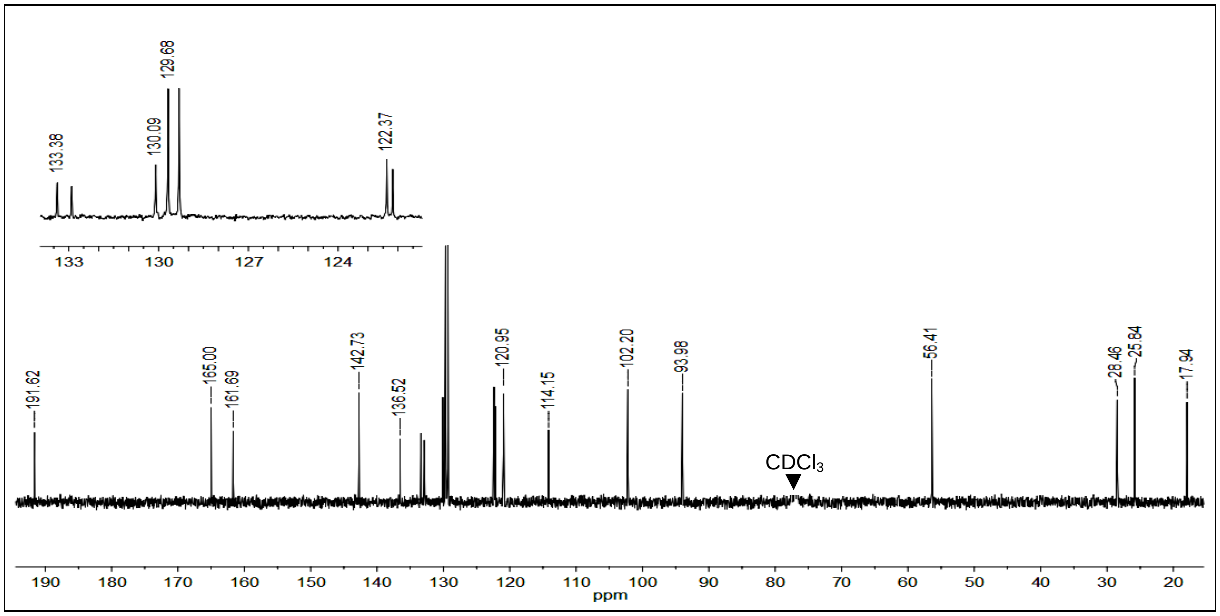

^{13}C NMR (**28**)

7-(methoxymethoxy)-2-(4-methoxyphenyl)-6-(3-methylbut-2-en-1-yl)-4H-chromen-4-one (**29**)

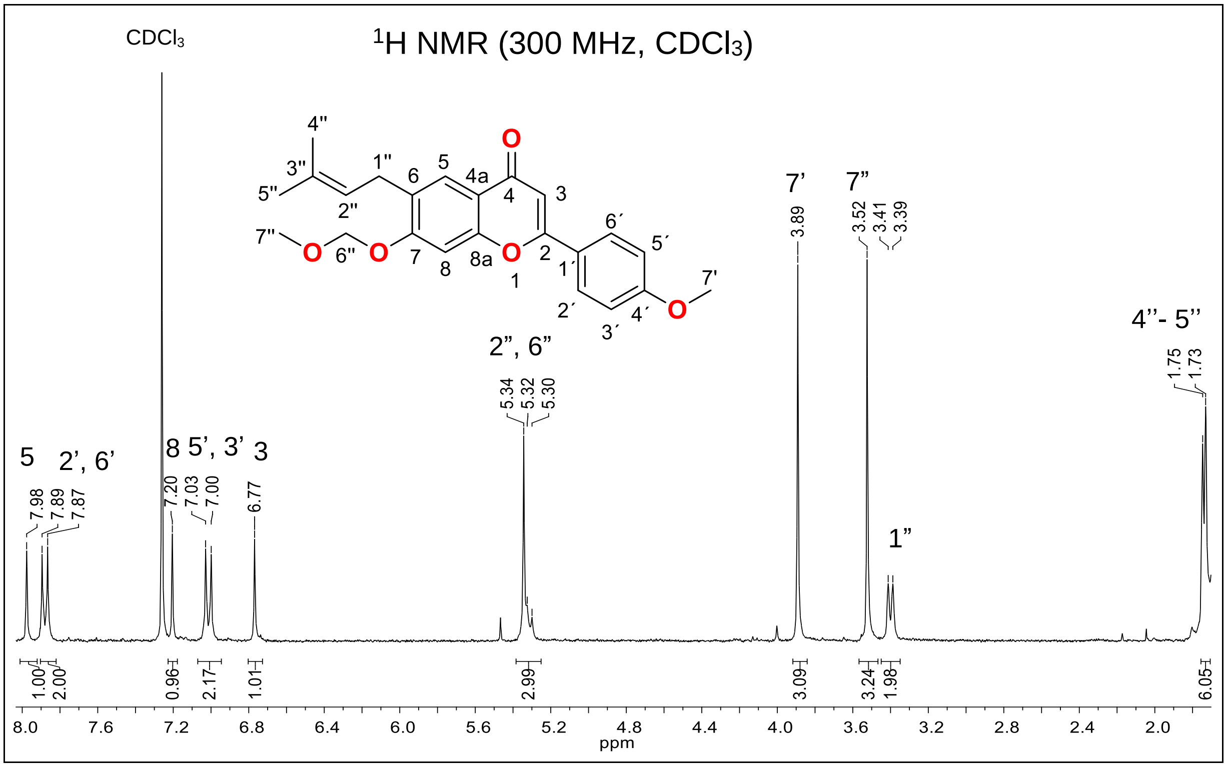

^{1}H NMR (**29**)

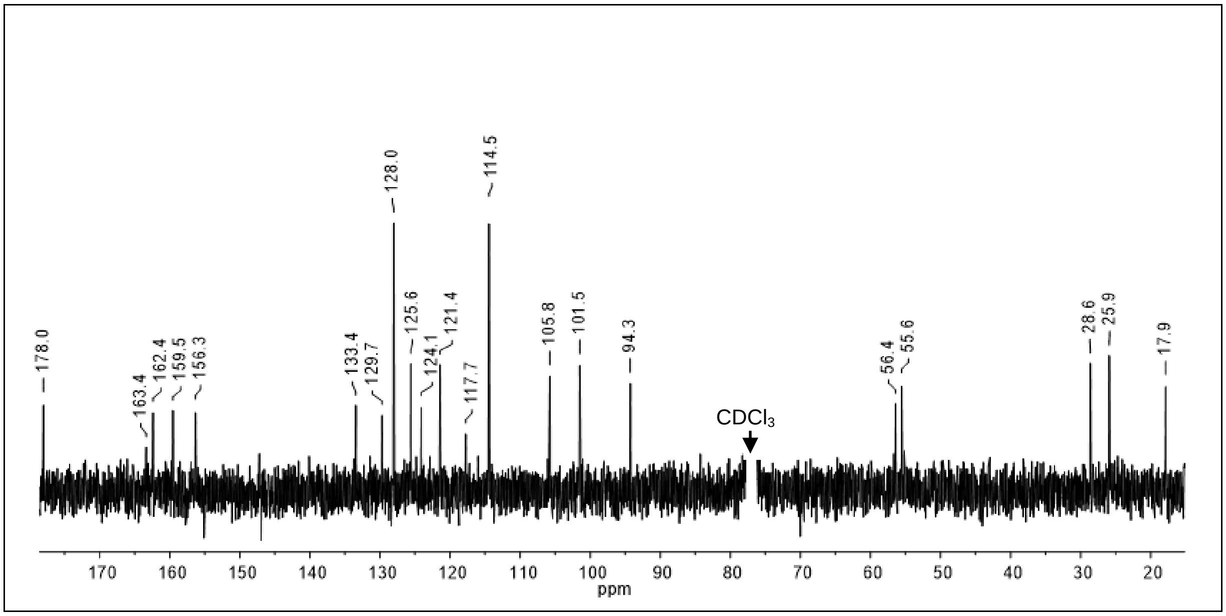

^{13}C NMR (**29**)

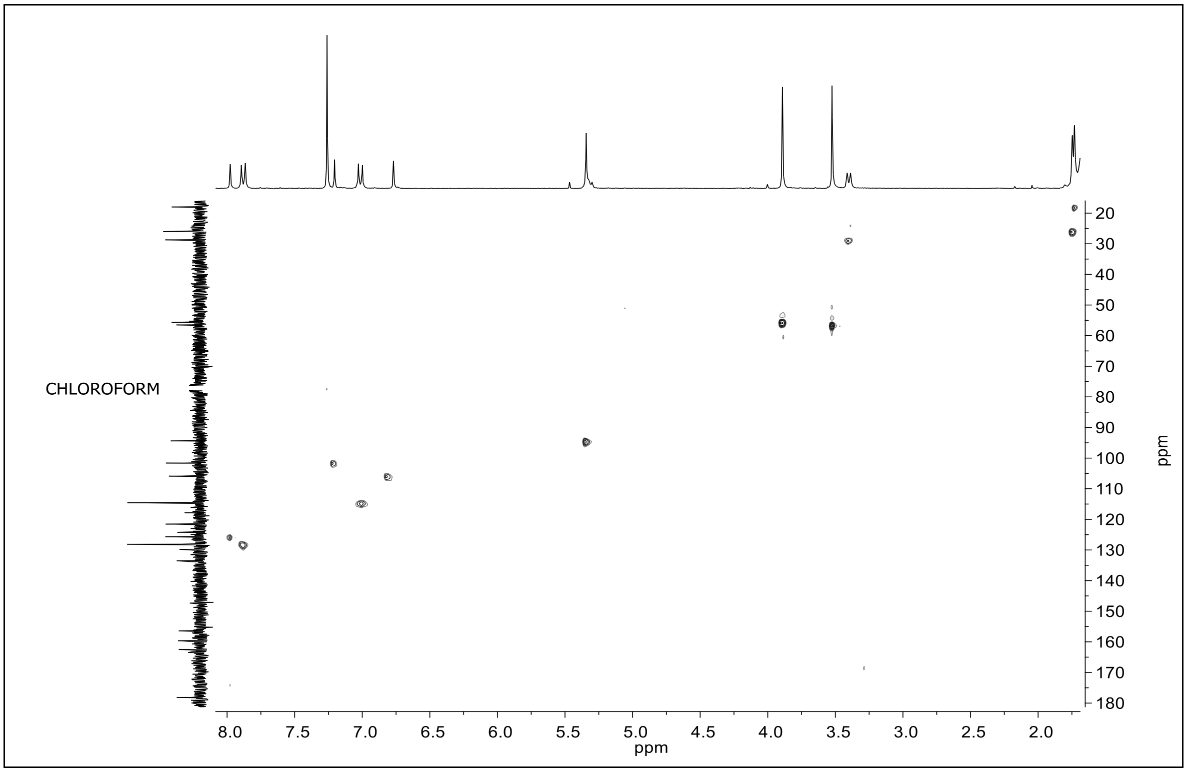

HMQC NMR (**29**)

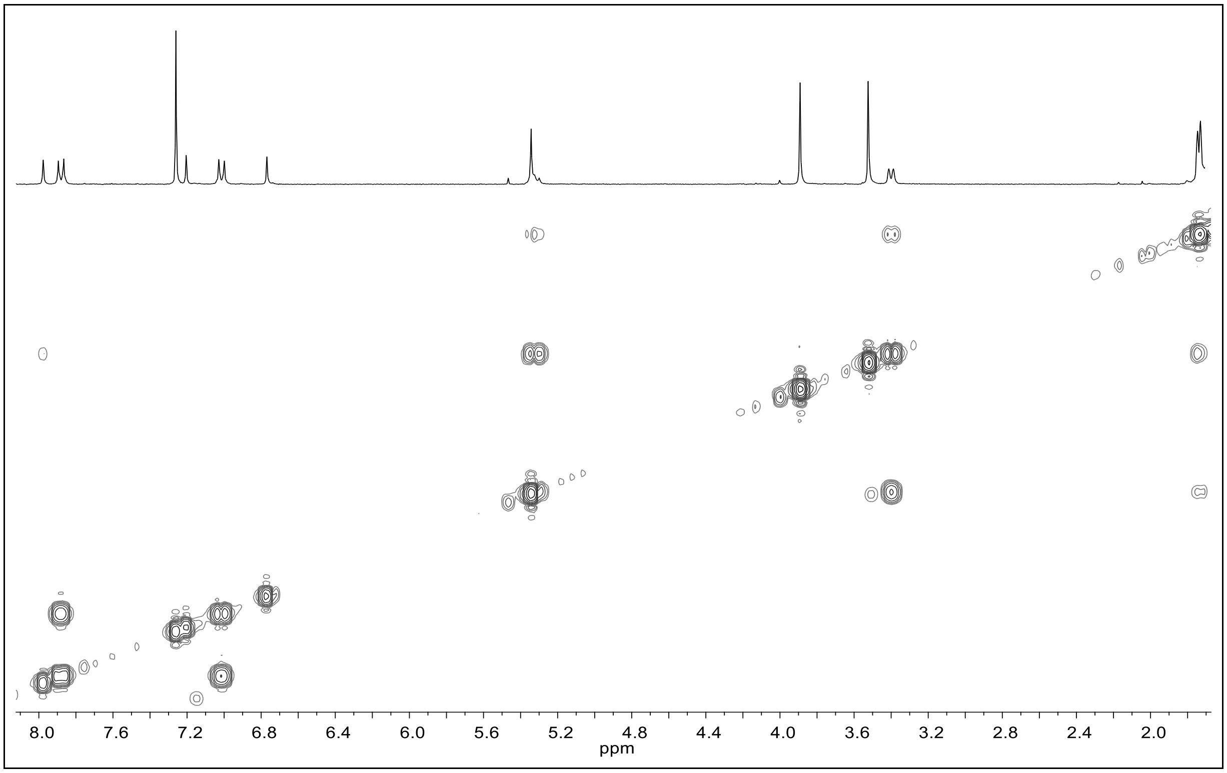

COSY NMR (**29**)

2-(4-chlorophenyl)-7-(methoxymethoxy)-6-(3-methylbut-2-en-1-yl)-4H-chromen-4-one (**30**)

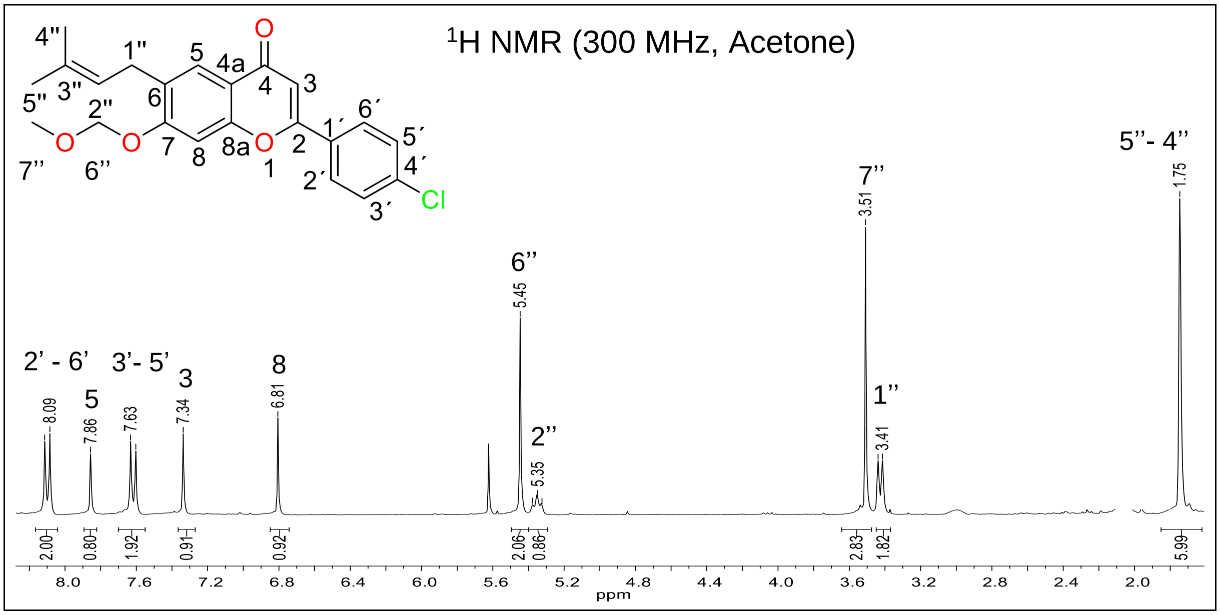

¹H NMR (**30**)

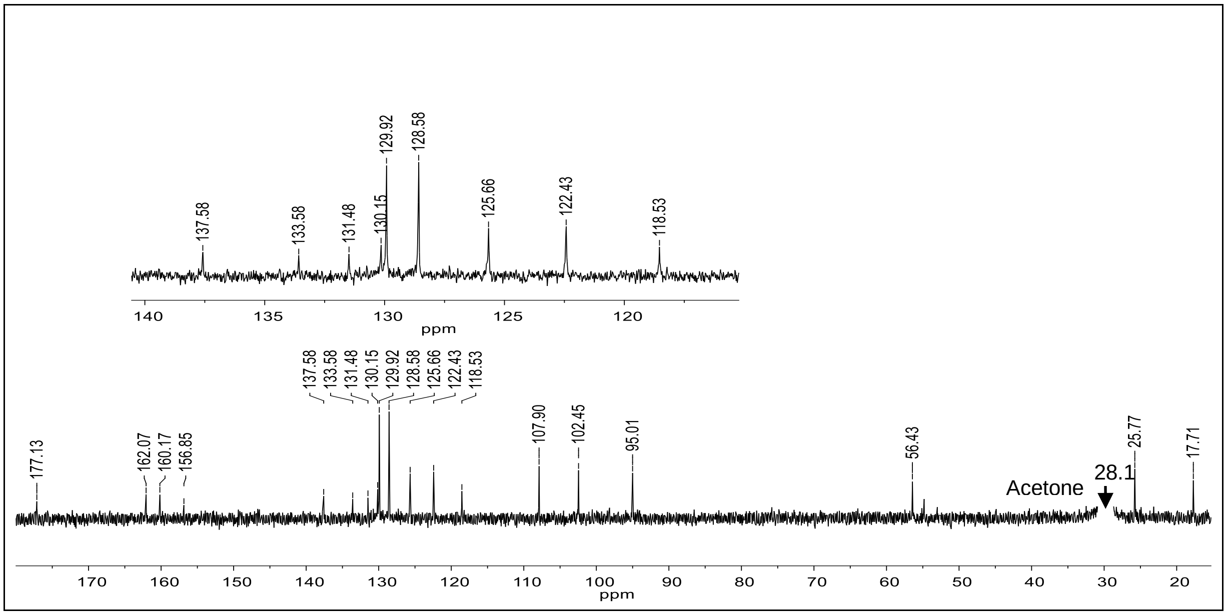

¹³C NMR (**30**)

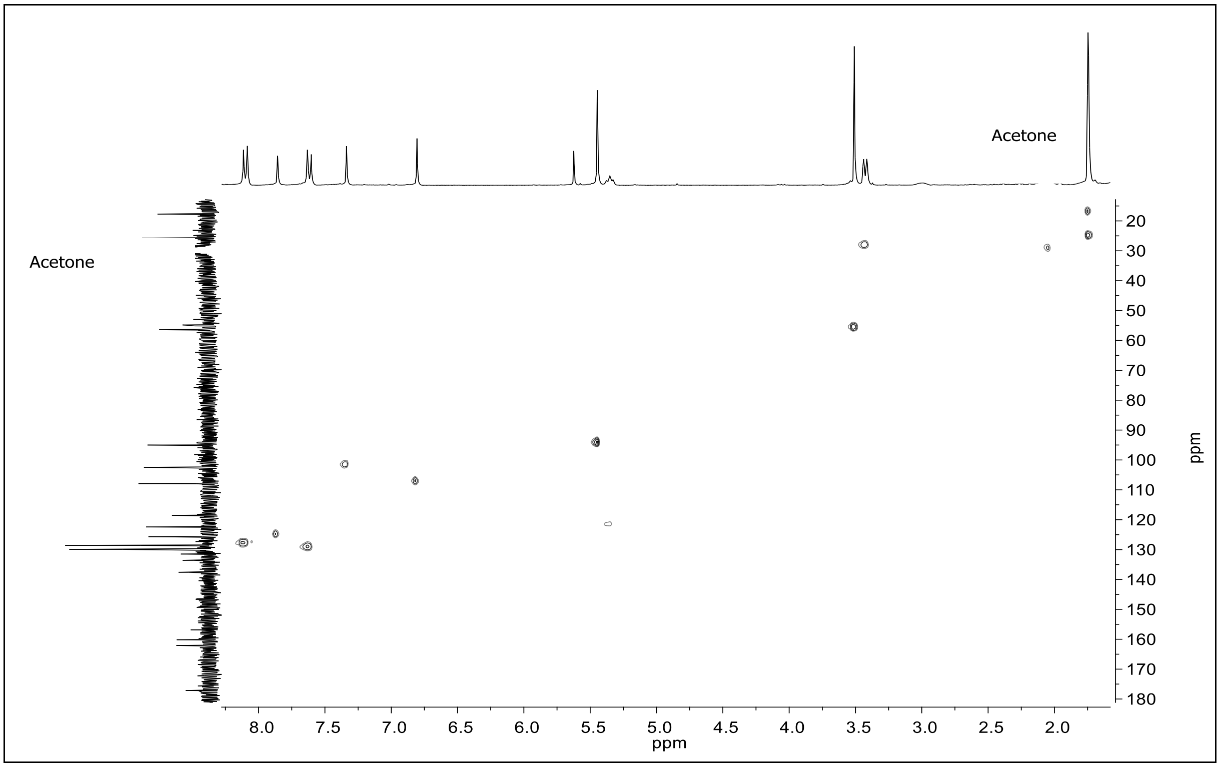

HMQC NMR (**30**)

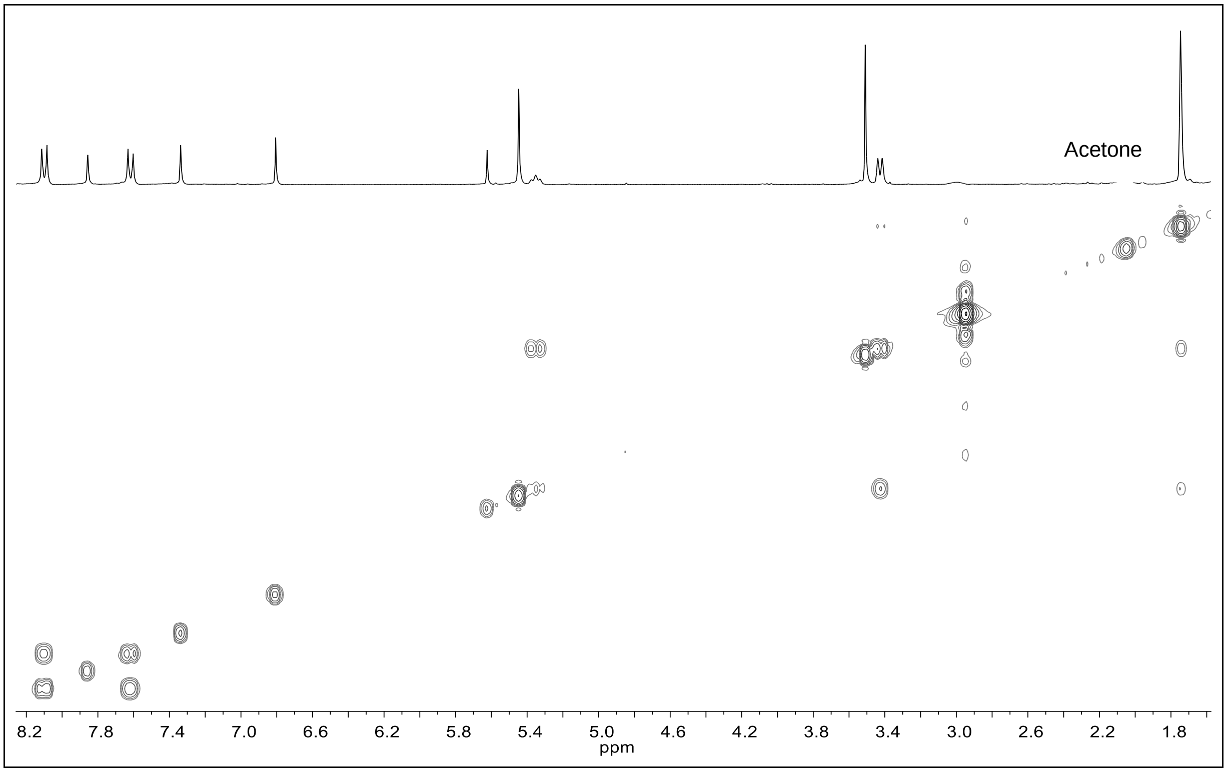

COSY NMR (**30**)
